# Targeting the major pro-inflammatory interleukin-6-type cytokine receptor gp130 by antagonistic single domain antibodies

**DOI:** 10.3389/fimmu.2025.1613004

**Published:** 2025-08-15

**Authors:** Silke Pudewell, Julia Heuser, Inna Dorogobed, Britta Lipinski, Thi Hong Hue Tran, Pia Metzenmacher, Richard Kunze, Felix Geyer, Stefan Zielonka, Doreen M. Floss, Harald Kolmar, Jens M. Moll, Jürgen Scheller

**Affiliations:** ^1^ Institute of Biochemistry and Molecular Biology II, Medical Faculty, Heinrich-Heine-University, Düsseldorf, Germany; ^2^ Applied Biochemistry, Institute for Organic Chemistry and Biochemistry, Technical University of Darmstadt, Darmstadt, Germany; ^3^ Biomolecular Immunotherapy, Institute for Organic Chemistry and Biochemistry, Technical University of Darmstadt, Darmstadt, Germany

**Keywords:** IL-6-type cytokines, glycoprotein 130, single domain antibody, inhibitor, inflammation

## Abstract

**Introduction:**

Although Interleukin (IL)-6-type cytokine signaling is critical for maintaining the body's homeostasis, aberrant signaling has been observed in numerous diseases including autoimmunity and cancer. Currently, all approved biologics that inhibit IL-6-type cytokines specifically target the key pro-inflammatory mediator IL-6 or its receptor (IL6R). Historically, direct inhibition of glycoprotein 130 (gp130)—the shared transmembrane receptor for IL-6-type cytokines—was avoided due to concerns that broad suppression might cause more harm than benefit. However, this view is being reconsidered in light of the clinical success of Janus kinase (JAK) inhibitors, which broadly disrupt cytokine signaling, including pathways mediated by gp130.

**Methods:**

Here we developed four single domain antibodies (sdAb), consisting out of a camelid-derived nanobody and a human Fc-fragment, and characterized them by direct protein interaction analysis, epitope binding, epitope binning, as well as inhibition of cytokine-induced stimulation and proliferation of appropriate Ba/F3 cell lines and trans-migration in HT-29 cells.

**Results:**

The four sdAb-Fc constructs GP01-, GP11- GP13- and GP20-Fc bind directly to gp130 in the cytokine binding module (CBM) and largely inhibit IL-6-type cytokine signaling by interfering with the high-affinity binding site of IL-6, IL-11, CLCF1, CT1, CNTF, OSM and LIF. Furthermore, we functionally demonstrate the inhibitory effect of the selected nanobodies in cell-based transmigration assays of the human colorectal cancer cell line HT-29.

**Discussion:**

In summary, our study has identified and characterized four novel inhibitory high-affinity gp130 nanobodies with potential for use in cytokine-dependent autoimmunity or cancer therapy.

## 1 Introduction

In the complex landscape of cellular communication, cytokines stand as pivotal signaling molecules orchestrating diverse physiological processes, from immune response modulation to tissue development and homeostasis. The trans-membrane β-receptor glycoprotein 130 (gp130) serves as a common signal transmitter for cytokines belonging to the interleukin (IL)-6-type cytokine family (1).

At its core, gp130 represents a transmembrane protein equipped with an extracellular and a cytoplasmic domain that receives and transmits signals upon cytokine binding. The versatility results from its association with a spectrum of different cytokines, including IL-6, IL-11, IL-27, leukemia inhibitory factor (LIF), oncostatin M (OSM), cardiotrophin-1 (CT-1), cardiotrophin-like cytokine factor 1 (CLCF1), and ciliary neurotrophic factor (CNTF) (2, 3). IL-6 and IL-11 are signaling *via* gp130 homodimers with the need of initial binding to membrane-bound or soluble IL-6 and IL-11 α-receptors (IL-6R, IL-11R) which is referred to as classic- or trans-signaling, respectively (4, 5). All other cytokines form signal-transducing receptor heterodimers consisting of gp130 and one of the β-receptors OSMR, LIFR or WSX-1.

Within the IL-6 family, binding of a cytokine to its receptors generally follows the same steps. The cytokine binds via site I to the α-receptor (IL-6R for IL-6, IL-11R for IL-11, CNTFR for CNTF and EBI3 for IL-27_p28). CLCF1, CT-1, LIF and OSM do not rely on α-receptor binding to interact with the β-receptor. Subsequently, all IL-6-type cytokines bind via site II to the first β-receptor. With the exception of IL-27 and IL-31, this initial high affinity interaction occurs with the cytokine binding module (CBM, domains 2 and 3 (D2/D3)) of gp130. For IL-27, the high affinity binding is formed by the CBM (D1/D2) of IL-27R. To form the active signaling receptor complex, these intermediate complexes recruit a second β-receptor via the low affinity binding site III of the cytokine. In detail, IL-6, IL-11 and IL-27 bind to D1 of gp130, CNTF, CT-1, CLCF1, LIF and OSM to D3/4 of LIFR and OSM to D2/3 of OSMR (5).

Through these partnerships, gp130 acts as a transmitter for extracellular signals to relay intracellular messages, triggering various downstream pathways such as the Janus kinase (JAK)-signal transducer and activator of transcription (STAT), extracellular signal-regulated kinase (ERK), and phosphoinositide 3-kinase (PI3K) pathways (6, 7). These signaling pathways result in the control of crucial biological responses ranging from immune regulation to hematopoiesis, neuronal survival and tissue repair.

Exploring the diverse roles of gp130-associated cytokines reveals their significance in both health and disease. Dysregulation of gp130 signaling has been implicated into many pathological conditions, including chronic inflammatory diseases, autoimmune disorders, and cancer, highlighting the therapeutic potential of targeting this signaling axis (8). To date, all approved biologics for blocking IL-6-type cytokines are directed against IL-6 or the IL-6R to selectively inhibit classic- and trans-signaling of the major pro-inflammatory cytokine IL-6 (9, 10). In addition, all other antibodies in pre-clinical and clinical development are also specifically directed against a single IL-6-type cytokine. These include the potentially anti-fibrotic and anti-aging IL-11 inhibitory antibody BI 765423, which has just entered Phase 1 (Clinical Trail ID: NCT05658107) (11, 12), and the potentially anti-cancer IL-27 inhibitory antibody SRF388 (Clinical Trail ID: NCT04374877) (13). Approaches to target other cytokines like LIF (14) and OSM (15), or the OSMR (16) did provide promising data for anti-tumorigenic effects, but have not yet reached clinical studies. Same for the small molecule sc144, which is inducing the internalization of gp130 and interfering with the phosphorylation of STAT3. This inhibitor is widely used in pre-clinical cancer studies but has not been approved in clinical studies to date (17–19). Another approach involves the development of a monoclonal antibody that disrupt gp130-mediated signaling, thereby attenuating downstream signaling and disease progression in a rheumatoid arthritis (RA) mouse model (20) or in myeloma growth (B-R3) (21). The overall severe phenotypes from global and conditional gp130 deficient mice (22) may suggest that targeting gp130 will have more deleterious than beneficial effects. However, this paradigm has generally been broken with the approval of small molecules broadly blocking cytokine-receptor associated JAKs. These inhibitors do not only interfere with gp130 receptor signaling but also with other cytokine receptor classes that also use JAKs as central kinases, including interferon-, erythropoietin-, and growth hormone-signaling. Already 10 JAK inhibitors have been approved by different healthcare authorities for the treatment of various diseases such as rheumatoid arthritis, ulcerative colitis, Crohn’s disease, Myelofibrosis and others (23–25).

Therefore, the development of first-in-class nanobodies that largely block gp130 signaling may be an attractive novel approach for future anti-inflammatory and anti-fibrotic therapies, additionally taking advantage of the manifold beneficial properties of single domain antibodies (sdAbs) (26–28). Moreover, inhibition of gp130 signaling holds potential in oncology, where aberrant cytokine signaling contributes to tumor progression and immune evasion (29–31). By dampening cytokine-driven tumor-promoting inflammation, gp130 inhibition may enhance the efficacy of cancer immunotherapy and sensitize tumors to conventional treatments (32). However, careful consideration of potential side effects, including immunosuppression and adverse effects on tissue homeostasis, is paramount in the development of gp130-targeted therapies to ensure their safety and efficacy in clinical settings (33). Ongoing research efforts aim to further elucidate the complexities of gp130 signaling and refine therapeutic strategies to harness its therapeutic potential while minimizing off-target effects.

Our study describes the development of camelid-derived antagonistic sdAbs directed against the CBM of gp130 with potential for therapeutic application in gp130-dependent diseases. Due to their mode of action, gp130 binders specifically inhibit IL-6-type cytokines that use site II for high-affinity binding of gp130, i.e., IL-6, IL-11, LIF, OSM and CNTF, but not IL-27.

## 2 Results

### 2.1 Development of heavy chain antibodies targeting gp130 from an immunized llama

**Figure 1:**
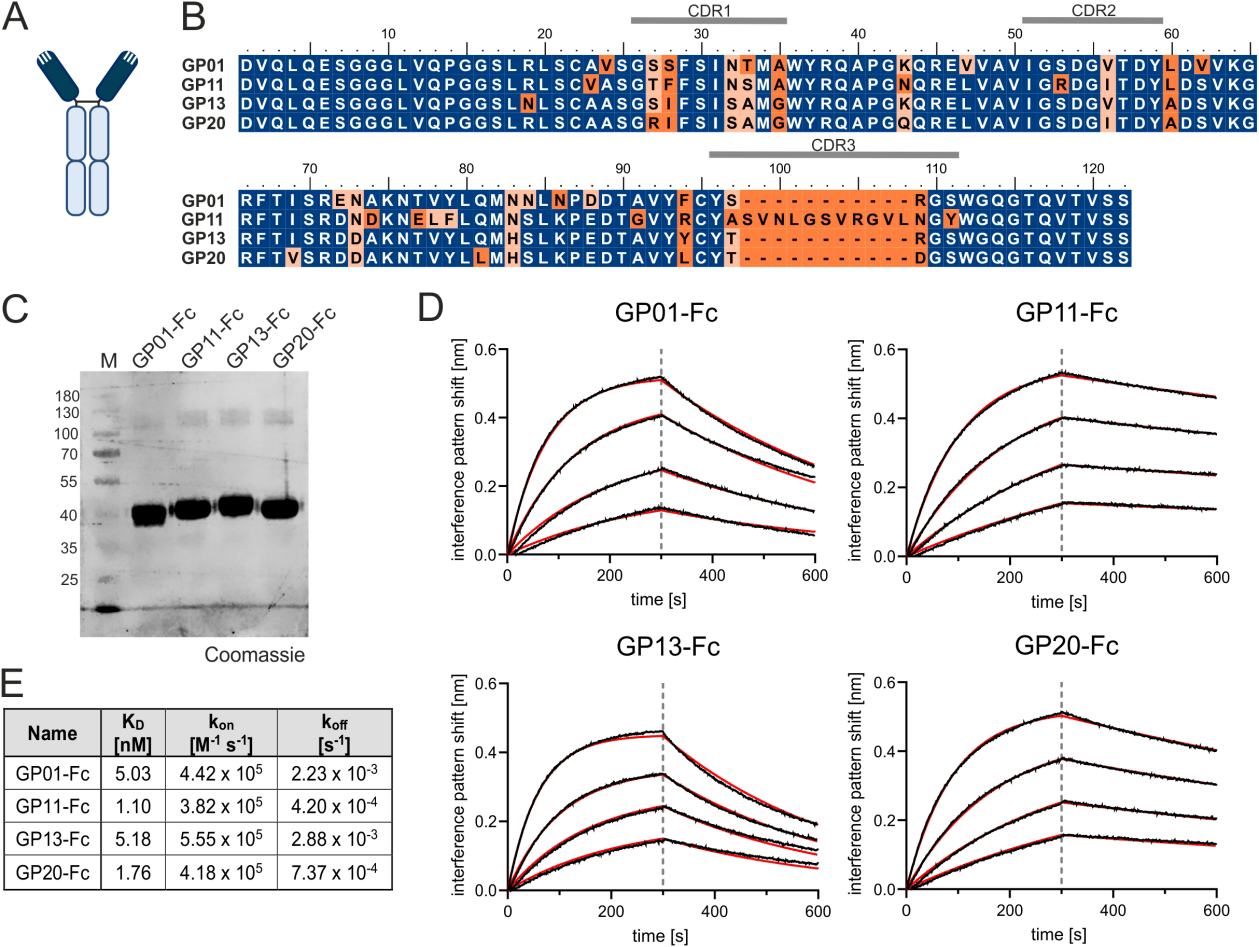
Characterization of gp130 nanobodies. **(A)** Structural organization a of single domain antibody. Dark blue color indicates the nanobody or VHH; the light blue color marks the Fc-region. **(B)** Alignment of GP01, GP11, GP13 and GP20. The Complementarity-determining regions (CDRs) are indicated above the sequence. Deviations are marked in orange. **(C)** Purified proteins GP01-Fc, GP11-Fc, GP13-Fc and GP20-Fc stained with Coomassie Brilliant Blue. **(D)** Association of 25 nM, 12.5 nM, 6.25 nM and 3.13 nM gp130-His (from top to bottom) to the four sdAbs with in BLI measurements. The data was fitted using a 1:1 binding model after Savitzky – Golay filtering (red lines). **(E)** Summery of the equilibrium dissociation constants (K_D_), as well as k_on_ and k_off_ rates of the BLI measurement.

The adaptive immune system of camelids comprises antibodies devoid of light chains referred to as heavy chain-only antibodies (hcAbs). In contrast to canonical antibodies composed of heavy and light chains, only a single variable domain of the heavy chain is required for antigen binding which is named variable domain of the heavy chain of a heavy chain-only antibody (VHH) or nanobody (dark blue, **Figure 1A**) (26). Due to their favorable properties including ease of production and high modularity, VHH domains are currently being exploited for various different scientific and therapeutical approaches (34). In this study, a llama was immunized with the recombinant human extracellular domain (ECD) of gp130 fused to the human Fc region. Subsequently, a VHH library was constructed based on total RNA and generated cDNA of the llama peripheral blood mononuclear cell repertoire using yeast surface display technology. Fluorescence-activated cell sorting enabled the enrichment of an antigen binding population (**Supplementary Figure S1A**). After sorting, sequencing revealed four clonotypes harboring seven unique sequences in total, referred to as GP01, GP06, GP11, GP12, GP13, GP14 and GP20 (**Supplementary Figure S1B**). The respective paratopes were genetically grafted onto the Fc region of an IgG1 backbone, expressed using Expi293™ cells, and purified by protein A chromatography (**Figures 1B, C**).

### 2.2 The four nanobodies GP01, GP11, GP13 and GP20 specifically target gp130 with high affinities

Further kinetic and biophysical properties of the seven gp130-interactors were determined by biolayer interferometry (BLI). Here, the Fc-tagged -nanobody fusion proteins were immobilized on an anti-hIgG Fc Capture (AHC) biosensor and titrated with increasing concentrations of gp130. Increasing layer thickness can be linked to protein-protein interaction. The negative control (only PBS), showed an increased layer thickness of about 0.2 nm, which could also be detected for the fusion proteins GP06-Fc, GP12-Fc and GP14-Fc. These nanobodies were therefore considered as non-binders and excluded from further experiments. (**Supplementary Figure S2**). The remaining for VHHs were analyzed further for their equilibrium dissociation constants (K_D_), as well as k_on_ and k_off_ rates, which revealed single-digit nanomolar affinity of GP01-Fc (5.03 nM), GP11-Fc (1.10 nM), GP13-Fc (5.18 nM) and GP20-Fc (1.76 nM) towards gp130 (**Figures 1D, E**). In order to predict their stability, the nanobodies were examined for their melting temperatures *via* a thermal shift assay, displaying temperatures of GP01: 62.1°C; GP11: 64.9°C; GP13: 68.7°C; GP20: 66.1°C, therefore considering these proteins as stable.

### 2.3 All four gp130 nanobodies bind within the D2 domain of gp130

**Figure 2:**
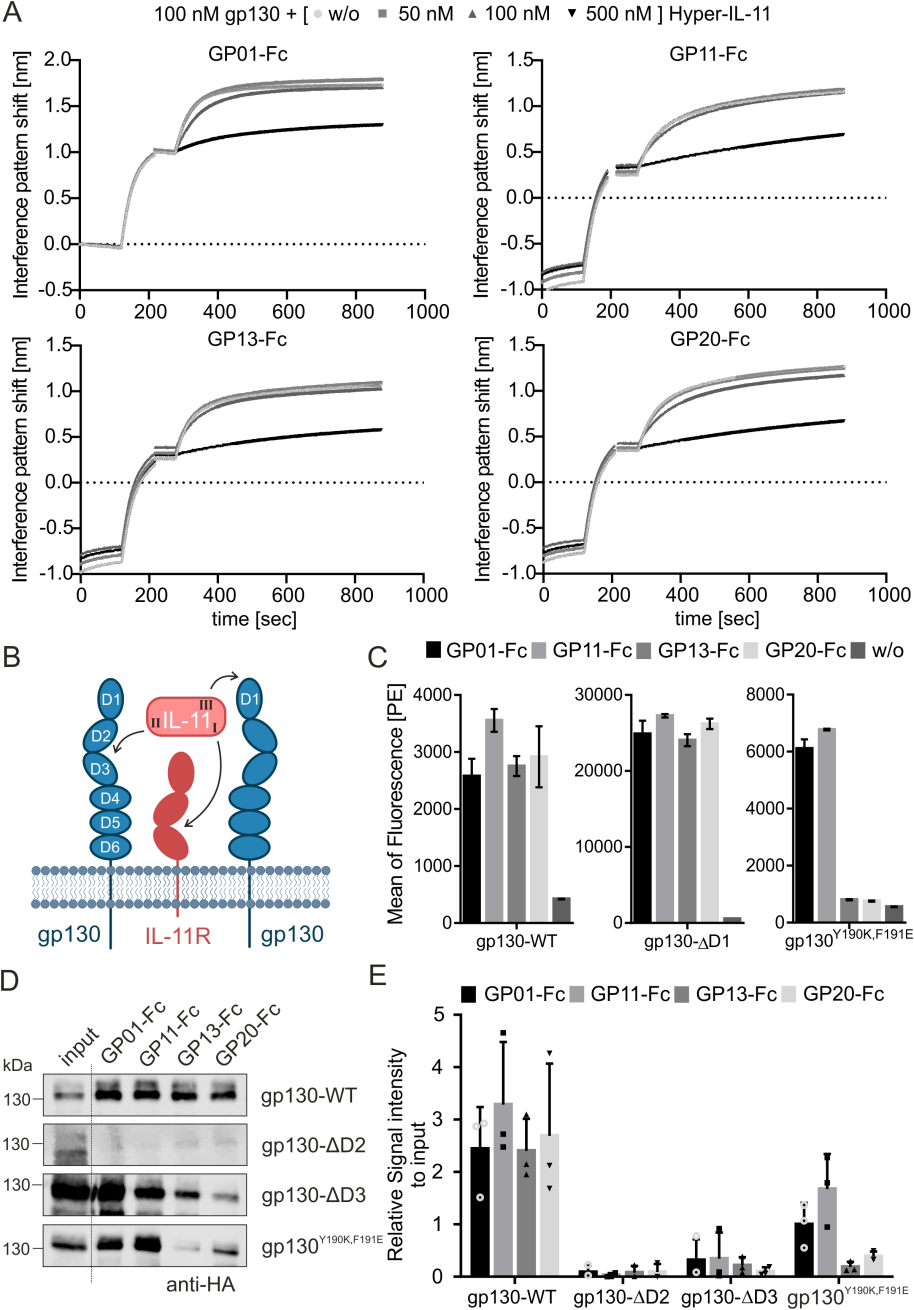
Epitope mapping of the gp130 nanobodies. **(A)** Competition assay of Hyper-IL-11 (IL-11:IL-11R fusion protein) with binding of the sdAbs to gp130 in BLI. GP01-Fc, GP11-Fc, GP13-Fc or GP20-Fc were coated on a an AHC biosensor. The interaction partner gp130 (100 nM) was preincubated with Hyper-IL-11 in increasing concentration of 50, 100 and 500 nM and measured in BLI for the association with the nanobody-Fc fusion protein. Please check **Supplementary Figure S3** for original data. **(B)** Graphical illustration of IL-11 binding to its receptors. First site I of L-11 associates with the α-receptor (IL-11R), next site II binds to the D2/D3 region of the β-receptor gp130, and finally site III binds to D1 domain of the second β-receptor gp130. The final complex is either formed by a tetramer or hexamer. **(C)** Immunofluorescence-based FACS analysis of sdAbs binding to gp130-WT, gp130-ΔD1 and gp130^Y109K,F191E^ variant, stable expressed in Ba/F3 cells. The secondary antibody against the Fc-part of the sdAbs is labelled with Phycoerythrin (PE). The data is represented as the mean of fluorescence in the PE-channel. Secondary antibody only without any sdAB (w/o) is used as an internal negative control. Untransduced Ba/F3 cells served as another negative control and are displayed in **Supplementary Figure S4**. (n=3) **(D)** Pull-down assay of gp130-WT, gp130-ΔD2, gp130-ΔD3 and gp130^Y109K,F191E^ variants (HA-tagged), overexpressed in HEK293T cells. Lysates were incubated with sdAbs and pulled down with Protein A breads. The proteins were analyzed by western blot using an anti-HA antibody. **(E)** Quantification of the pull-down analysis of three independent experiments, normalized to the signal intensity of the input. (n=3).

Next, the remaining four gp130-binders were analyzed regarding their binding sites, as well as their ability to compete with cytokine association. This approach was carried out by another BLI experiment, coating the VHH-Fc fusion proteins to the AHC biosensor and pre-incubating the interaction partner gp130 with increasing concentrations of an IL-11:IL-11R-fusion protein (Hyper-IL-11). In accordance with their high sequence identity (**Figure 1B**), all gp130-specific nanobodies were not able to bind simultaneous to Hyper-IL-11 and are therefore competing for either the same or an overlapping binding epitope as the cytokine-receptor-complex (**Figure 2A**, **Supplementary Figure S3**).

The association of a cytokine to its receptors is generally similar within the IL-6-type cytokine family and is coordinated by the same binding sites as previously described (**Figure 2B**). The specific α- and β-receptor combinations are listed in **Table 1**. Since the four nanobodies are blocking the association of Hyper-IL-11 to gp130, it is reasonable to assume that the binding epitope of all four nanobodies may lay within the D1-D3 domain of gp130. To verify and pinpoint the specific binding epitopes, protein association and pull-down assays were carried out using a set of gp130 deletion variants: gp130-ΔD1 lacking domain 1, gp130-ΔD2 lacking domain 2 and gp130-ΔD3 lacking domain 3 as well as the gp130 wild type (WT) as a positive control. Additionally, gp130^Y190K,F191E^ was used, as these specific mutations in the D2 domain are known to play a crucial role in the direct binding of IL-6 and IL-11 (PBD: 8D82) (35, 36).

**Table 1:** Cytokine binding sites.

Cytokine	IL-6	IL-11	OSM	LIF	CNTF	p28 (IL-27)
Site I α-receptor	IL-6R	IL-11R	–	–	CNTFR	EBI3
Site II β-receptor	gp130 (D2/D3)	gp130 (D2/D3)	gp130 (D2/D3) low affinity	gp130 (D2/D3) high affinity	gp130 (D2/D3) high affinity	WSX-1
Site III β-receptor	gp130 (D1)	gp130 (D1)	OSMR high affinity	LIFR low affinity	LIFR	gp130 (D1)

The association of the four nanobodies with gp130-WT, gp130-ΔD1 and gp130^Y190K,F191E^, which were expressed in Ba/F3 cells, was detected using an immunofluorescence-based FACS analysis. Gp130-WT and gp130-ΔD1 could bind to all nanobodies, indicated by an increase in the mean of fluorescence (**Figure 2C**, **Supplementary Figure S4**). The gp130^Y190K,F191E^ variant could only bind to GP01 and GP11. Based on this data, the D1 domain of gp130 was excluded as the binding epitope. Next, binding to the domains D2 and D3 were investigated via pull-down assay. The binding epitope of D2 domain was further validated by using the gp130^Y190K,F191E^ mutant for pull-down analysis. After cell lysis, the variants were incubated with the Fc-tagged nanobodies and precipitated by Protein A agarose beads (**Figures 2D, E**). Strong binding was observed for gp130-WT. The gp130-ΔD2 variant was not precipitated by the nanobodies. Gp130-ΔD3 was precipitated by all nanobodies, but with decreased efficacy. Interestingly, the two nanobodies GP13-Fc and GP20-Fc still displayed weak association with the gp130^Y190K,F191E^ mutant. Whereas GP20-Fc exhibits very attenuated binding, GP13-Fc seems to fail association almost completely. The amino acids Y190 and F191 of gp130 form direct contacts with site II of IL-6 and are in close proximity to the site II contact site of D3 domain (F191 (D2) and V252 (D3) - 7.87 Å (**Supplementary Figure S5**, PDB:8D82 (36)). Our data point to an epitope for GP13-Fc and GP20-Fc including Y190 and F191 in the EF-loop of the D2 domain and maybe part of the D3 domain. Docking analysis were performed using AlphaFold 3 and confirmed a binding epitope in the transition of the D2 to D3 domain for all four nanobodies, including a close proximity of F191 with the nanobodies GP13 and GP20 (**Supplementary Figure S6**). Moreover, the competition of the four nanobodies was tested to check whether they could bind simultaneously to gp130. Therefore, epitope binning analysis were performed by BLI measurements. The data underlines that all four nanobodies block each other from their binding sites and cannot bind simultaneously to gp130 (**Supplementary Figure S7**). Taken together, the data suggest that the binding epitope of GP01-Fc and GP11-Fc mainly lies within the D2 domain of gp130 but outside of the amino acids Y190 and F191, and a binding epitope which encompasses the EF-loop that connects D2 and D3 domain including Y190 and F191 for GP13-Fc and GP20-Fc.

### 2.4 The nanobodies GP01-Fc, GP11-Fc, GP13-Fc and GP20-Fc block the signaling of gp130 site II-binding IL-6-type cytokines

Binding of the gp130 nanobodies to the D2 or D2/D3 domain, and competing with Hyper-IL-11 for the same binding site suggested inhibitory properties for gp130. Most, but not all, IL-6-type cytokines use site II to bind to gp130, and signaling of those cytokines may be impaired. IL-6-type cytokine-induced cellular proliferation as well as STAT3 and ERK phosphorylation were analyzed using Ba/F3 cells expressing the respective IL-6-type cytokine receptor combinations (**Table 2**).

**Table 2:** IC_50_s of gp130 nanobodies.

Cytokine	Ba/F3 Receptors	IC_50_ [nM]			
		GP01	GP11	GP13	GP20
IL-11	Gp130 IL-11R	2.64 ± 1.49	1.88 ± 1.54	5.35 ± 3.89	9.94 ± 4.02
IL-11 IL-11-R	Gp130	5.49 ± 4.24	5.01 ± 3.10	9.50 ± 7.10	19.32 ± 10.13
IL-6	Gp130 IL-6R	–	–	–	–
Hyper-IL-6 (IL-6/IL-6R fusion protein)	Gp130	0.43 ± 0.31	0.52 ± 0.10	0.59 ± 0.40	3.43 ± 1.86
OSM	Gp130 OSMR	10.45 ± 15.73	4.68 ± 4.18	10.48 ± 11.71	20.11 ± 21.77
LIF	Gp130 LIFR	47.83 ± 17.81	24.22 ± 8.18	78.45 ± 24.93	127.18 ± 76.00
CNTF	Gp130 CNTFR LIFR	–	–	–	–
mIL-27 (p28/EBI3 fusion protein)	Gp130 WSX-1	No inhibition	No inhibition	No inhibition	No inhibition
Hyper-mIL-23 (p40-p19 fusion protein)	Gp130 IL-12Rβ1 IL-23R	No inhibition	No inhibition	No inhibition	No inhibition

IC_50_ ± SD (n=3)/- not to be calculated.

**Figure 3:**
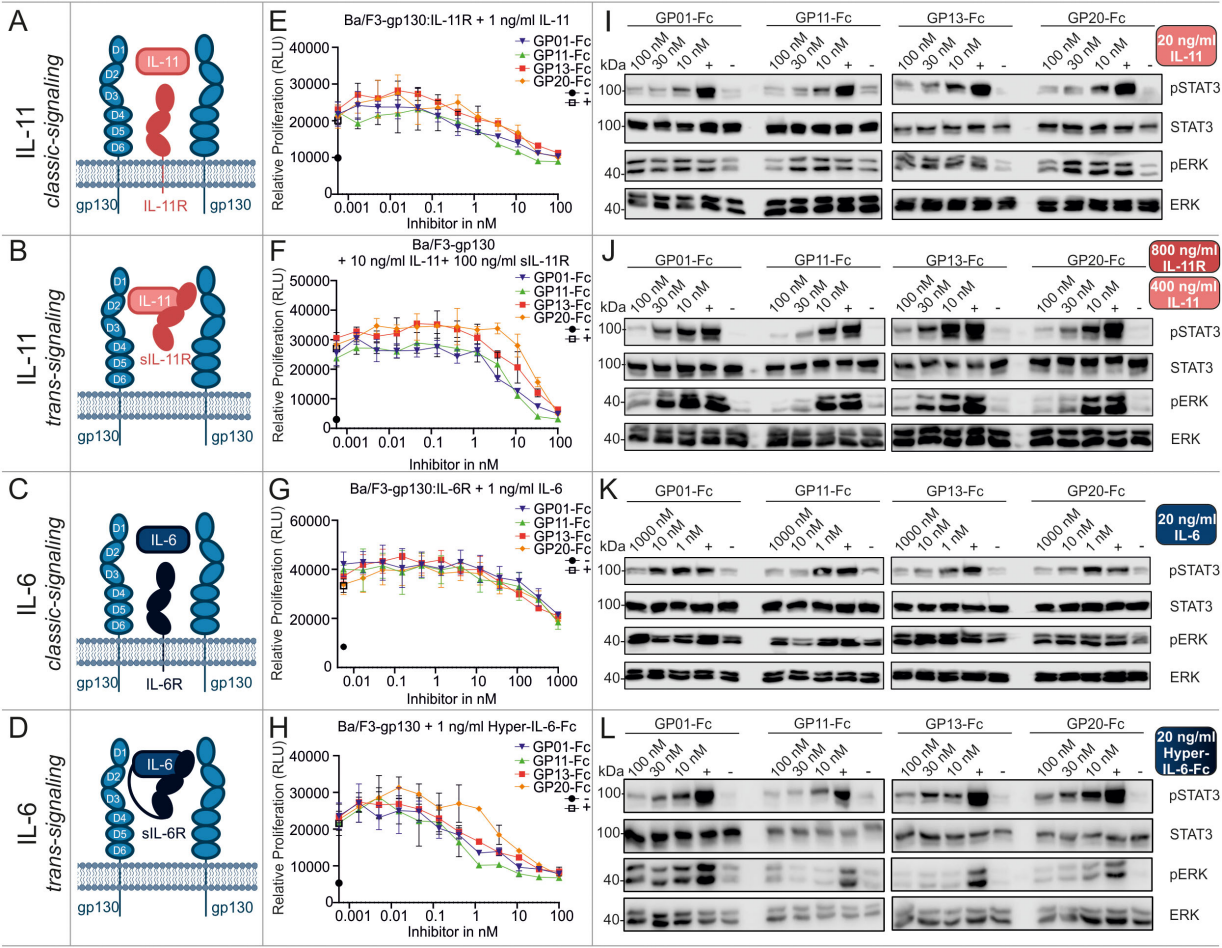
Biological characterization of the sdAbs towards IL-11 and IL-6 classic and trans-signaling. **(A–D)** Graphical illustration of signaling components. Displayed receptors are overexpressed on Ba/F3 cells and used for the respective proliferation and stimulation assays. (**E, F)** Proliferation assays of Ba/F3 cells stimulated with 1 ng/ml IL-11 (IL-11 classic-signaling), 10 ng/ml IL-11 and 100 ng/ml sIL-11R (IL-11 trans-signaling), 1 ng/ml IL-6 (IL-6 classic-signaling), or 1 ng/ml Hyper-IL-6-Fc (IL-6 trans-signaling). The gp130 nanobodies were titrated to the cells in a concentration between 1000 and 0.00056 nM as indicated (▼GP01-Fc, ▲GP11-Fc, ◼ GP13-Fc, • GP20-Fc). The negative control (-) black circle •, represents unstimulated cells, the positive control (+) white square □, displays cells treated with cell supernatant containing approx. 20 ng/ml Hyper-IL-6. Every value is measured in triplicates and all assays are performed in three independent experiments (n=3). **(I–L)** Stimulation assays of Ba/F3 cells expressing the respective receptors and being stimulated with 20 ng/ml IL-11 (IL-11 classic-signaling), 400 ng/ml IL-11 and 800 ng/ml sIL-11R (IL-11 trans-signaling), 20 ng/ml IL-6 (IL-6 classic-signaling), or 20 ng/ml Hyper-IL-6-Fc (IL-6 trans-signaling). The sdAbs were added in a concentration of 10, 30 and 100 nM or 1, 10 and 1000 nM as indicated. The negative control (-) represents unstimulated cells, the positive control (+) displays cells treated with cell supernatant containing approx. 20 ng/ml Hyper-IL-6. The lysates were blotted against pSTAT3^Y705^, total STAT3, pERK^T202,Y204^, and total ERK. The blots are representative for at least three independent experiments.

We first performed cell biological assays for IL-11 and IL-6 classic- and trans-signaling, comprising the membrane bound or the soluble α-receptor, respectively (**Figures 3A–D**). The proliferation assays (**Figures 3E–H**), as well as the results for phospho-STAT3 and -ERK levels (**Figures 3I–L**) revealed a strong inhibitory effect of all gp130 nanobodies towards cell proliferation and downstream signaling. For IL-11, classic- and trans-signaling were both strongly inhibited, whereas IL-6 trans-signaling was much more affected than IL-6 classic-signaling. The overall half maximal inhibitory concentrations (IC_50_) of the nanobodies are shown in **Table 2**. Remarkably, ERK signaling was less responsive when stimulated by classic-signaling (especially IL-6), which is also reflected in poor regulation upon inhibitor treatment.

**Figure 4:**
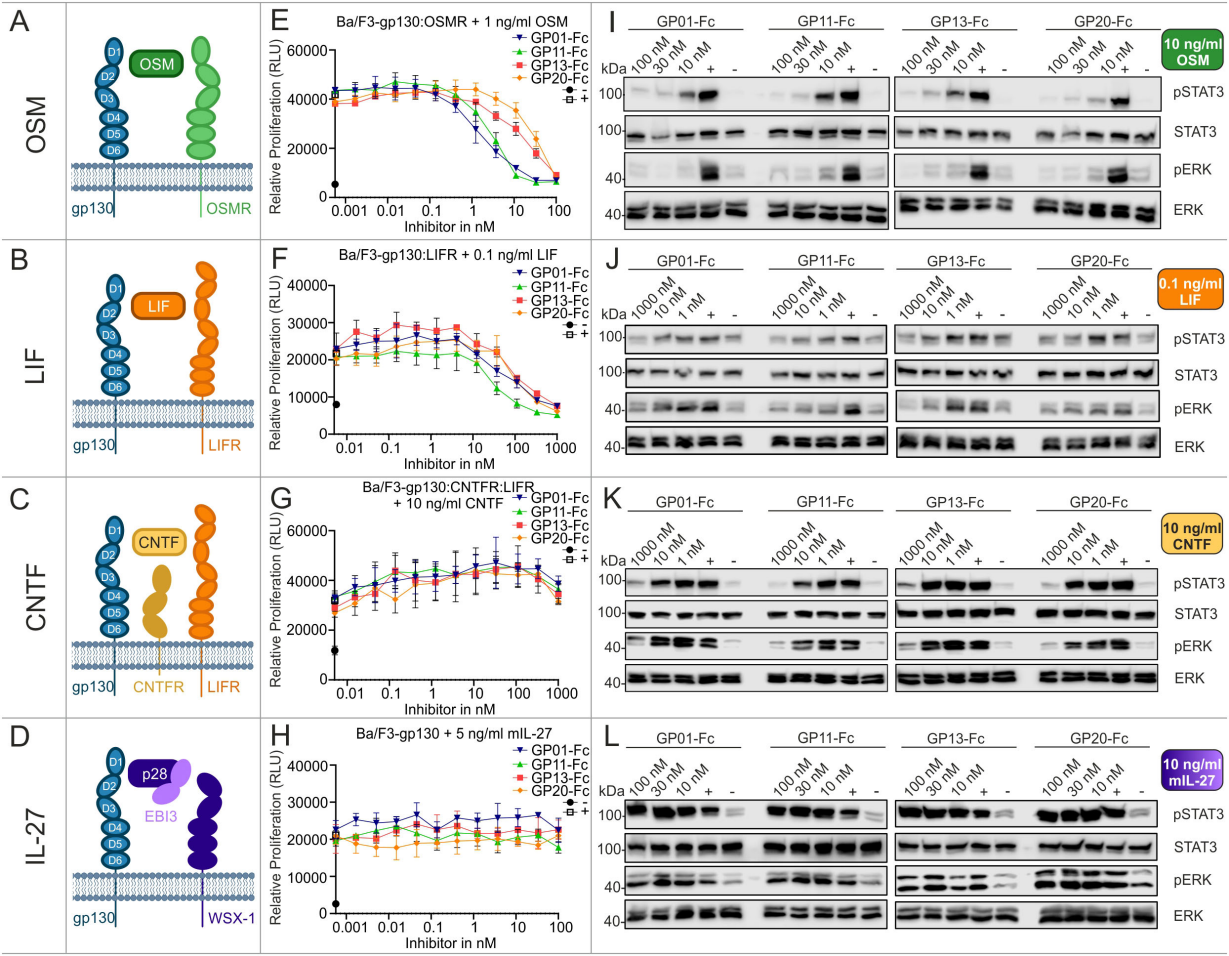
Biological characterization of the sdAbs towards OSM, LIF, CNTF and IL-27 signaling. **(A–D)** Graphical illustration of signaling components. Displayed receptors are overexpressed on Ba/F3 cells and used for the respective proliferation and stimulation assays. **(E, F**) Proliferation assays of Ba/F3 cells stimulated with 1 ng/ml OSM, 0.1 ng/ml LIF, 10 ng/ml CNTF, or 5 ng/ml mIL-27. The gp130 nanobodies were titrated to the cells in a concentration between 1000 and 0.00056 nM as indicated (▼GP01-Fc, ▲GP11-Fc, ◼ GP13-Fc, • GP20-Fc). The negative control (-) black circle •, represents unstimulated cells, the positive control (+) white square □, displays cells treated with cell supernatant containing approx. 20 ng/ml Hyper-IL-6. Every value is measured in triplicates. All proliferation assays are performed in three independent experiments (n=3). **(I–L)** Stimulation assays of Ba/F3 cells expressing the respective receptors and being treated with 10 ng/ml OSM, 0.1 ng/ml LIF, 10 ng/ml CNTF, or 10 ng/ml mIL-27. The sdAbs were added in a concentration of 10, 30 and 100 nM or 1, 10 and 1000 nM as indicated. The negative control (-) represents unstimulated cells, the positive control (+) displays cells treated with cell supernatant containing approx. 20 ng/ml Hyper-IL-6. The lysates were blotted against pSTAT3^Y705^, total STAT3, pERK^T202,Y204^, and total ERK. The blots are representative for at least three independent experiments.

The signaling pathways of OSM, LIF, CNTF and IL-27, also utilize the gp130 receptor for transducing intracellular signaling cascades (**Figures 4A–D**). The experimental setup used to investigate these pathways regarding their ability to be inhibited by the gp130 nanobodies was analogue to the previous measurements and was based on proliferation assays (**Figures 4E–H**), as well as the results for phospho-STAT3 and -ERK levels (**Figures 4I–L**). Of the four cytokines, OSM was most effectively inhibited by the gp130 nanobodies. LIF inhibition was achieved at higher nanobody concentrations. CNTF-induced cell proliferation was also only slightly reduced at high concentrations of the gp130 nanobodies, but reduced STAT3 and ERK phosphorylation was observed at a nanobody concentration of 1 µM. Interestingly, IL-27-induced signaling and proliferation was not inhibited by any of the nanobodies at any concentration. Combining these results with the binding epitope of the nanobodies studied in **Figure 2** this was expected, because site II of IL-27 interacts with WSX-1 and only site III associates with gp130 via the D1 (**Table 1**).

To exclude some off-target effects of the nanobodies, the closely related IL-23, which utilizes the gp130-related cytokine receptors IL-12Rβ and the IL-23R to induce intracellular signaling, was used as a negative control. As shown in **Supplementary Figure S8**, the four nanobodies did not prevent Ba/F3-gp130:IL-12Rβ1:IL-23R cell proliferation and/or STAT3/ERK phosphorylation.

Summarizing the biological effects of the gp130 nanobodies regarding gp130-dependent signaling pathways, underlines the inhibitory effect of GP01-Fc, GP11-Fc, GP13-Fc and GP20-Fc. GP11-Fc did overall show the lowest IC_50_ values and can therefore be considered as the most potent inhibitor. Based on the proliferation data, IL-6 trans-signaling is inhibited best, in contrast to classical IL-6 signaling, whose inhibition is rather weak. IL-11 and OSM signaling is both inhibited quite well, followed by LIF, which displays about 5-times higher IC_50_ values. CNTF signaling was barely affected, even though high concentrations did have an effect in stimulation assays.

### 2.5 No crosstalk with murine gp130

**Figure 5:**
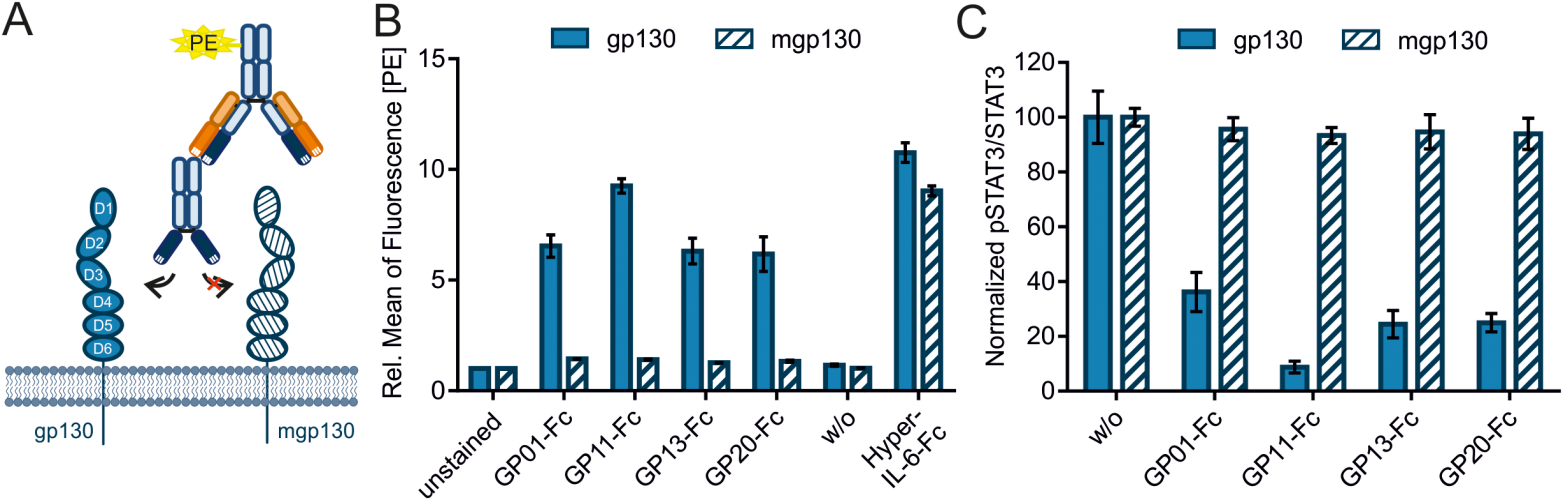
Evaluating cross talk of the sAbs with murine gp130. **(A)** Graphical illustration of the immunofluorescence-based binding assay of the nanobody-Fc fusion proteins to gp130, detected by a PE-labeled anti-human-Fc antibody. **(B)** Ba/F3 cells either expressing human gp130 (filled bars) or murine gp130 (mgp130, striped bars) were incubated with 3µg of the sdAbs. Binding was detected by the PE-labeled secondary antibody targeting the Fc-Tag of the sdAbs. Secondary antibody only (w/o) is used as a negative control. Hyper-IL-6-Fc (300 ng for gp130 and 3 µg for mgp130) was used as a positive control. The data is normalized to unstained cells and represented as the relative mean of fluorescence in the PE-channel. (n=3) **(C)** Phosphorylation of STAT3^Y705^ was measured via intracellular STAT3 staining using FACS. Ba/F3 cells were either stimulated with 20 ng/ml (gp130) or 200 ng/ml (mgp130) Hyper-IL-6-Fc and spiked with 1000 nM GP01-Fc, GP11-Fc, GP13-Fc or GP20-Fc. Cells without sdAb (w/o) were used as a negative control. Results are displayed as the mean of fluorescence of pSTAT3^Y705^ in Alexa647 channel divided by the mean of fluorescence of total STAT3 in the PE channel. The values are normalized to the unstimulated cells (not shown) and the negative control (w/o). (n=3).

Most studies related to specific diseases and their treatment involve mouse experiments. Therefore, the cross-reactivity of the nanobodies against the murine gp130 receptor (mgp130) was investigated in an immunofluorescence-based binding assay (**Figure 5A**). Ba/F3 cells that either express the human or the murine gp130 receptor were incubated with the nanobodies and detected by an PE-labeled anti-human-Fc-antibody via FACS analysis. The experiment could not show any binding of the nanobodies to mgp130 (**Figure 5B**, n=3). Hyper-IL-6-Fc was used as a positive control. The data was normalized to the unstained cells. Next, phosphorylation of STAT3 at Y705 was measured by intracellular STAT3 staining. The cells were pre-incubated with the sdAbs and stimulated with Hyper-IL-6-Fc. Human gp130 signaling was decreased by the nanobodies, in contrast to murine gp130 signaling, which could be induced by human Hyper-IL-6-Fc but was not inhibited by GP01-Fc, GP11-Fc, GP13-Fc or GP20-Fc (**Figure 5C**, n=3).

### 2.6 Gp130 nanobodies inhibit Hyper-IL-11 induced transmigration in HT-29 cells

**Figure 6:**
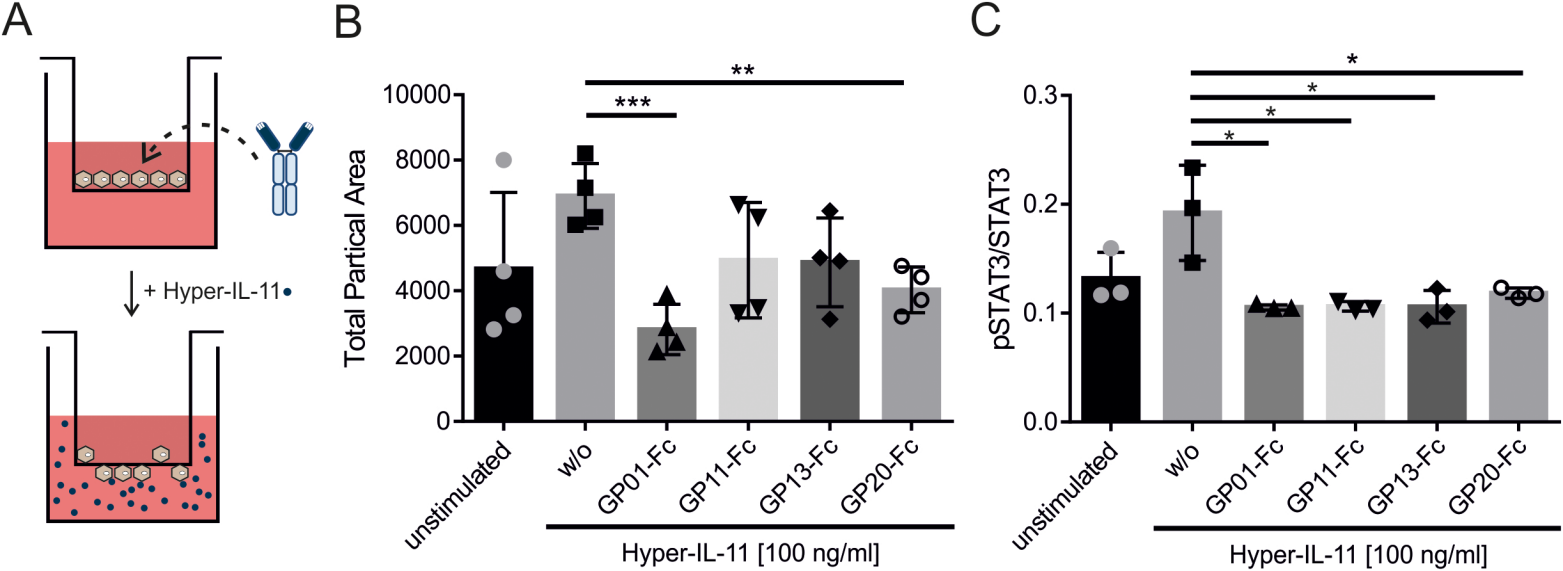
Determining the effect of sdAbs on the Hyper-IL-11 induced transmigration of HT-29 cells. **(A)** Graphical illustration of the transmigration experiment. HT-29 cells and nanobody-Fc fusion proteins were placed in the transwell insert. Hyper-IL-11 was added to the lower buffer. **(B)** Transmigration was induced by 100 ng/ml Hyper-IL-11. The sdAbs were added in a concentration of 100 nM. Unstimulated cells served as a negative control, cells without any sdAB (w/o) served as a positive control. Migrated cell were analyzed by removing cells on top of the membrane and staining the remaining cells with crystal violet. The membranes were imaged under a microscope with 2x magnification and analyzed using ImageJ software. The results are displayed as total particle area, which represent migrated cells. Statistics were added by GraphPad Prism using an unpaired t test. The statistical analysis revealed p-values of w/o *vs*. GP01-Fc p=0.0006; GP11-Fc p=0.0994; GP13-Fc p=0.0520; GP20-Fc p=0.0031. (n=4) **(C)** Phosphorylation of STAT3^Y705^ was measured via intracellular STAT3 staining using FACS. HT-29 cells were stimulated with 100 ng/ml Hyper-IL-11 and spiked with 100 nM GP01-Fc, GP11-Fc, GP13-Fc or GP20-Fc. Cells without sdAb (w/o) were used as a positive control. Unstimulated cells were utilized as a negative control. Results are displayed as the mean of fluorescence of pSTAT3^Y705^ in Alexa647 channel divided by the mean of fluorescence of total STAT3 in the PE channel. (n=3). (*p < 0.05; **p < 0.01; ***p < 0.001).

Transmigration of the colorectal cancer cell line HT-29 was described earlier to be IL-11 dependent (37). To examine our nanobodies in a functional setting, the transmigration of HT-29 cells was aimed to be inhibited by blocking IL-11 signaling. Therefore, HT-29 cells were plated in a trans-well together with the nanobodies, and placed in a medium containing Hyper-IL-11 (**Figure 6A**). After 5 hours, transmigration was checked by removing cells on top of the membrane and staining migrated cells on the lower side with crystal violet. The data shows a Hyper-IL-11 dependent transmigration of HT-29, which was reduced by the gp130-nanobodies (**Figure 6B**). GP01-Fc and GP20-Fc showed the best inhibitory effect in this setting, followed by GP11-Fc and GP13-Fc. The pSTAT3^Y705^ levels of HT-29 in response to Hyper-IL-11 stimulation were checked via intracellular pSTAT3/STAT3 staining using FACS. All four nanobodies could reduce the pSTAT3^Y705^ levels to the level of the unstimulated cells (**Figure 6C**).

## 3 Discussion

Targeting the signaling cascade of IL-6-type cytokines has proven to be a promising approach for therapies against autoimmune diseases, various cancers and other cytokine-related syndromes. Selective inhibition of a single signaling cascade has long been preferred to minimize off-target effects. With the approval of a variety of JAK inhibitors, the focus shifted from local to global inhibition of these signaling cascades. Although JAK inhibitors are approved for specific therapies, adverse effects such as infections, cytopenia and hyperlipidemia are common (25, 38). The main task in blocking and fine-tuning these signaling cascades in therapeutic terms is to find the balance between specific and global inhibition. Gp130 as a β-receptor for almost all IL-6-type cytokines could establish the balance between specific α-receptor or cytokine and non-specific, global JAK inhibition (39).

Inhibition of gp130 was suggested for example, for cancer associated cachexia. Here, not only IL-6 but also LIF and OSM play a role in the rapid loss of adipose and skeletal muscle tissue (40). Moreover, the general inhibition of gp130 for cancer therapy has been intensively discussed, as a large number of different types of cancer exhibit aberrant production and response of IL-6-type cytokines that can trigger cell proliferation, differentiation, migration and invasion [Reviewed in (39)]. In rheumatoid arthritis, not only the dysregulation of the immune response, especially by the proinflammatory cytokine IL-6, has a serious impact on the progression of the disease, but also the differentiation of osteoclasts and their high expression of gp130, both of which can be reduced by inhibiting gp130 (20, 41). Clinical applications need careful patient stratification and combination strategies with other immune-modulating agents, especially in complex diseases like cancer and autoimmunity.

A handful of monoclonal antagonistic antibodies against gp130 have been reported and tested in specific disease settings. The first, named B-R3, was published 1996 and mainly interfered with the dimerization of gp130 but, except for OSM, not with the cytokine binding. In 2017, it was again reported to be a promising agent for inhibiting myeloma cell growth, interestingly reducing the phosphorylation of STAT3 but not ERK in response to IL-6 and LIF stimulation. Nevertheless, treatment with B-R3 prevented tumor formation in a xenograft model using SCID mice (21). Another mAb called M10 was successfully used in an RA mouse model, therefore displaying cross reactivity with murine gp130. However, the inhibition of other IL-6-like cytokines was not investigated further (20).

Nanobodies compared to monoclonal antibodies have many advantages. As their binding paratope comprises only three instead of six hyper variable regions (HVRs) on one, instead of two variable domains, these proteins are able to bind to smaller and/or harder accessible binding epitopes. Furthermore, nanobodies generally do not forfeit any binding affinity compared to mAbs, but are more stable in terms of temperature, pH and proteolytic degradation (34, 42). One of the main advantages in research might be their simple structure that allows genetic engineering like multimerization or fusion with other nanobodies, proteins or even antibodies to create multivalent proteins. The fusion to an IgG Fc-Tag for instance, increases the clearance time in serum, however a shorter half-life might be favorable in short term applications like the treatment of a cytokine storm (43). In a clinical setting, nanobodies are highly requested due to their small size and the associated ability to penetrate e.g., solid tumors, but also their high target affinity (28). In 2018, the first nanobody-based drug, named Ceplacizumab, was approved by the European Medicines Agency for the treatment of thrombotic thrombocytopenic purpura. Since then, three other nanobody based therapeutics were approved: Envafolimab, Ozoralizumab and Ciltacabtagene autoleucel, paving the way for a whole set of novel drugs (34, 44).

The binding affinities of our four nanobodies GP01, GP11, GP13 and GP20 to gp130 are between 1.1-5.18 nM and are therefore within the same range or even slightly better than other monoclonal antibodies on the market targeting IL-6 like cytokine signaling. For instance, HZ0408b or Siltuximab, both IL-6 antibodies, have an affinity of about 1.075 nM and 11.68 nM respectively (45). The novel IL-6R antibody HZ0412a and Tocilizumab, the latter being already approved by FDA and used in patients with, for example, rheumatoid arthritis, display an K_D_ of 14.5 nM and 38.8 nM respectively (46). All K_D_s mentioned were measured by BLI experiments with a similar setup and are therefore suitable for comparison. Functionally wise, the IC_50_s of the nanobodies need to be taken into account. For IL-6 trans-signaling, IC_50_s range between 0.43 to 3.43 nM, measured in Ba/F3-gp130 cells. Tocilizumab displays an IC_50_ of around 0.27 nM in the same setup (47), which is within a similar range. The apparently much weaker ability of the nanobodies to further inhibit classical IL-6 signaling compared to IL-6 trans-signaling may be an additional advantage, as classical signaling is more associated with maintenance of the basal immune response, which is for example impaired upon JAK inhibitor treatment, and trans-signaling is associated with strong immune overactivation and pro-inflammatory events in certain diseases.

Additional to the K_D_ the specific association and dissociation of the nanobodies can have an impact on their effectivity. Whereas a fast association is needed to compete with the cytokine-receptor complex for the binding at the D2 domain, the dissociation should be rather slow, in order to increase the dwell time of the inhibitor. On the other hand, a slow dissociation could trigger internalization of the receptor-inhibitor-complex and therefore the removal of the latter from the system (48, 49). Our nanobodies all exhibit a comparable fast association but differ in their dissociation from the receptor. GP01 and GP13 display a quite fast dissociation, whereas GP11 and GP20 bind comparable strong to their target and dissociate more than 10-times slower. The result of these differences in a cellular system needs to be investigated experimentally and can be hardly predicted.

The binding epitopes of all four nanobodies are located in the D2 domain and are therefore within the CBM of the receptor. This could be substantiated as the nanobodies compete with the binding of the IL-11/IL-11R (Hyper-IL11) complex. The initial association of IL-11 and gp130 is predominantly driven by the amino acids W142, F147 and F169, all located within the D2 domain of gp130 (50). The amino acids Y190 and F191, which were mutated in this study to further pinpoint the binding epitope, are important for the structural formation of gp130. These amino acids in the EF-loop of gp130 D2 domain, interact with V252 in the BC-loop of the D3 domain, consequently keeping the receptor in a non-ligand bound, inactive state (51). Different variants of deletions in gp130 between V184 and V196 can be found in several cancer patients disturbing the D2-D3 interactions, and probably keeping the receptor in a primed state, which is easier to be activated or even constitutive active (52) (COSMIC Database (COSG3869)). Since we hypothesize the nanobodies GP13 and GP20 to bind to the region of the EF-loop, these nanobodies might stabilize the inactive state of the receptor, which could be favorable in a cellular system. This could be the reason why GP20, the nanobody with the highest IC_50_ values in Ba/F3 cells, is very effective in inhibiting the transmigration of HT-29 cells induced by Hyper-IL-11.

The lack of cross-reactivity of our nanobodies with murine gp130 presents a limitation for conventional *in vivo* validation. This is a common challenge in the development of human-specific biologics targeting non-conserved epitopes. As a result, standard mouse models cannot be used to assess efficacy or pharmacokinetics directly. To overcome this, several strategies can be employed: (i) generating surrogate nanobodies that target murine gp130 with similar binding properties to their human counterparts; (ii) conducting initial proof-of-concept studies in *ex vivo* human tissue models or organoids, which offer mechanistic insights while minimizing species-specific confounders; or (iii) using humanized mouse models expressing human gp130, which allow for a more physiologically relevant evaluation of therapeutic activity. Each of these approaches has its trade-offs in terms of complexity, cost, and translational relevance, but they are crucial steps toward advancing gp130-targeted nanobody therapies into clinical development.

It should be noted that murine IL-6 is not cross reactive with human IL-6R:gp130, but can induce signaling to human gp130 by trans-signaling via the murine IL-6:sIL-6R complex (53). Murine LIF is not cross-reactive to human LIFR:gp130 complex, but human LIF can bind to murine LIFR (54). IL-11 however can bind and can be bound by either human and murine cytokine:receptor combination (55, 56). Regardless of which cytokine is chosen, these cross-reactive relationships must be taken into account.

Humanized liver models in mice for example, can be used to investigate the IL6-gp130 axis on acute phase activation, but also lipid droplet accumulation by expression of murine IL-6R in the human liver cells (57, 58). For investigating anticancer properties, SCID mice, can be introduced to multiple myeloma (MM), patient derived xenograft (PDX), or tumor samples. These mice can for example express human IL-6 (NOD.Cg-*Prkdc^scid^ Il2rg^tm1Sug^* Tg(CMV-IL6)1-1Jic/JicTac) or other human cytokines and being investigated for the activity of the gp130 nanobodies. General xenograft models in immunodeficient mice can be used to study tumor growth and invasion of cells dependent on IL-6-type cytokines when produced by the tumor itself and stimulated in an autocrine and paracrine manner. Since there is no cross-reactivity with murine gp130, our inhibitors should be exclusively active against human tumor cells.

In order to adapt the nanobodies to new approaches, including *in vivo* models, it is possible to create bi- or multivalent fusion proteins, which target for example other cell specific antigens. Deleting the Fc-domain might decrease serum half-life, but could help in terms of drug delivery for solid tissues (59). These modifications will be customized to the respective therapeutic or scientific issue.

All in all, our study presents four new global inhibitors against IL-6-type cytokines that utilize the D2/D3 domain of gp130 for interaction, including: IL-6, IL-11, LIF, OSM and CNTF. We were able to establish a conclusive epitope mapping and binding affinity analysis and furthermore tested the inhibitory effect of these sdAbs in a cell-based proliferation and stimulation assay as well as the inhibition of transmigration of the IL-11-dependent human colon carcinoma cell line HT-29.

### 3.1 Limitations of this study

While our findings are based on established cell line models, which offer reproducibility and mechanistic insight, we acknowledge that validation in primary cells or *ex vivo* systems would further enhance physiological relevance. Future studies will be needed to assess potential toxicity and evaluate translational applicability in more complex biological systems.

## 4 Methods

### 4.1 Cells, proteins and reagents

Ba/F3 and HEK293T cells were obtained from the Leibnitz Institute DSMZ-German Collection of Microorganisms and Cell Culture (Braunschweig, Germany). Phoenix Eco cells, used for retroviral transduction, were obtained from Ursula Klingmüller (DKFZ, Heidelberg, Germany). HT-29 cells were kindly provided by AG Georg Flügen (UKD, Düsseldorf). ExpiCHO™ and Expi293™ cells were purchased from Thermo Fisher Scientific (#A29127, #A14635, Waltham, USA),. Ba/F3, HEK293T and Phoenix Eco cells were cultured in Dulbecco’s modified Eagle’s Medium (DMEM) high glucose culture medium (GIBCO, Life Technologies, Darmstadt, Germany), containing 10% fetal calf serum (FCS) (GIBCO, Life Technologies, Darmstadt, Germany), 60 mg/l penicillin (P) and 100 mg/l streptomycin (S) (1% P/S) (Genaxxon Bioscience GmbH, Ulm, Germany). Growth medium of Ba/F3 or Ba/F3-gp130 cells additionally include 0.2% of conditioned medium from IL-3 secreting WEHI-3B murine myelomonocytic leukemic cells (ACC 26, DSMZ), or a stable clone of CHO-K1 cells secreting Hyper-IL-6 respectively (stock solution approximately 10 µg/ml as determined by ELISA). HT-29 were cultured in McCoy’s 5A medium containing 2 mM L-Glutamine (VWR, Radnor. USA), 10% FCS and 1% P/S. All cells were cultured in a water-saturated atmosphere incubator at 37°C with 5% CO_2_. ExpiCHO™ and Expi293™ cells were cultured in ExpiCHO™ expression medium or Expi293™ medium in an orbital shaker (135 rpm), at 37°C and 8% CO_2_. Recombinant human OSM (catalog no.295-OM), LIF (catalog no. 7734-LF), and CNTF (catalog no. 257-NT) were purchased from R&D Systems (Minneapolis). Recombinant human IL-6 (catalog no.170-076-104) was purchased from Miltenyi Biotec GmbH (Bergisch Gladbach). Hyper-IL-6, IL-11 and mIL-27 were expressed as soluble proteins in Expi293™ and purified via affinity chromatography as described before (60, 61). sIL-11R was obtained from Bio-Techne (Wiesbaden, Germany).

Primary antibodies in this study include: STAT3 ((79D7), #4904), phospho-STAT3^Tyr705^ ((D3A7), #9145), p44/42 MAPK (ERK 1/2) (#9102) and phospho-p44/42 MAPK^Thr202/Tyr204^ (pERK 1/2) (#4370) from Cell Signaling Technology (Danvers, USA), STAT3 (#560391) and (p)STAT3^Tyr705^ (#557815) (1:200) (BD Biosciences, Franklin Lakes, NJ, USA). Secondary antibodies include: IRDye^®^ 800 CW anti-Rabbit IgG (#926-32213) and IRDye^®^ 680 RD anti-Mouse IgG (#926-68070) from LI-COR (Lincoln, USA) and the R-Phycoerythrin (PE) labelled anti-human-Fc antibody (#109-115-098, Jackson Immuno Research, Baltimore).

### 4.2 Lama immunization and cDNA generation

A lama (Lama glama) was immunized with gp130 extracellular domain fused to human Fc (47) at preclinics GmbH, Potsdam, Germany. All experimental procedures and animal care were in accordance with local animal welfare protection laws and regulations. In brief, for each immunization 300 μg protein diluted in a volume of 1 ml PBS were emulsified with either 1 ml Complete Freund’s Adjuvant (first immunization) or Incomplete Freund’s Adjuvant (subsequent immunizations). Injections were administered subcutaneously at three sites. A total of four immunizations were performed over the course of 56 days (0, 28, 42, and 56). On day 60, blood (100 ml) was collected and total RNA was extracted. After the study, camelids remained alive. mRNA, isolated from peripheral B cells of the immunized Lama was transcribed to cDNA utilizing SuperScript III Reverse Transcriptase (Invitrogen) as described previously (62, 63).

### 4.3 Yeast surface display library screening

PCR amplification of VHH encoding genes and generation of a yeast display library was performed essentially as described in (64). Briefly, the VHH coding sequences were transferred into pCT and pTTH shuttle vectors, that differ in the orientation of the VHH gene to the gene for Aga2p that mediates surface anchoring (65). Both libraries, pTTH and pCT, were mixed together, 2*10^8^ cells were sorted in the first round and 50,000 cells in the following rounds two and three, after preincubation with 250 nM of biotinylated soluble human gp130. Surface presentation was verified using a mouse anti-c-myc antibody and APC conjugated anti-mouse antibody (Invitrogen, 1:50) for c-myc detection and PE coupled streptavidin (Invitrogen, 1:50) for proof of gp130 binding. After the third enrichment round, plasmids were isolated from single clones and the nanobodies were reformatted via Golden Gate Assembly into vector pTT5-Fc, which contains a human IgG1 Fc. The proteins were expressed using Expi293™ or ExpiCHO™ cells and purified by Protein A affinity chromatography.

### 4.4 Transfection and protein expression in Expi293™ or ExpiCHO^TM^

Protein expression in Expi293™ or ExpiCHO™ cells was carried out following the manufactures protocol using the Expression System Kit (#A14635, #A29133, Thermo Fisher Scientific, Waltham, USA). In brief, cells were splitted to reach an exponential growth phase. Cells were transfected by ExpiFectamine™ and OptiPRO medium with the gene of interest cloned in a pcDNA3.1or pTT5 vector. For secreted proteins, this vector must contain a C-terminal signal peptide that enables secretion. 20 hours post transfection, Enhancer and Feed reagents were added to the culture to improve protein production. The cells were cultured on the orbital shaker for up to 10 days before harvesting the supernatant.

### 4.5 Protein purification

Protein purification of Fc-tagged proteins are performed using a Protein A MabSelect™ HiTrap™ columns (GE Healthcare, Chalfront St Giles, UK) and the ÄKTA Start system with the UNICORN™ start 1.3 software (Cytiva, Marlborough, USA). The cell culture supernatant of Expi293™ or ExpiCHO™ cells were centrifuged twice and filtered through a 0.45 µm pore size membrane. The ÄKTA Start, as well as the ProteinA column was washed with PBS before loading the filtered supernatant. The flowrate of all steps was set to 1 ml/min. Afterwards, the column was washed with PBS and citrate buffer with a pH of around 5. The Fc-tagged proteins were eluted using a citrate buffer with pH 3.5. The eluate’s pH was neutralized by adding Tris-buffer with a pH of 9, aiming for a pH between 6.5 and 7.5. Following, NAP™-25 columns (GE Healthcare, Chalfront St Giles, UK) were used to exchange the buffer to PBS (pH 7.5). Samples were concentrated using Amicons with an appropriate molecular weight cutoff (Merck, Darmstadt, Germany) and the concentration was determined with the NanoDrop 2000c (Thermo Fisher Scientific, Waltham, USA) at 280 nm, taking into account the corresponding extinction coefficient. The purity of the proteins was checked in an SDS-polyacrylamide gel after electrophoresis and staining with Coomassie. The gels were documented using the LI-COR Odyssey^®^ XF Imaging System (Lincoln, USA) and the Image Studio software version 5.2.

### 4.6 Biolayer interferometry

For BLI measurements, the Octet RED96 system (ForteBio, Pall Life Science) with 25°C and 1000 rpm agitation settings was used. To evaluate binding affinities 3 µg/mL of the single domain antibodies in PBS were loaded on anti-hIgG Fc capture (AHC) biosensors for 180 s, followed by 60 s sensor rinsing in kinetic buffer (KB; PBS + 0.1% Tween-20 and 1% bovine serum albumin (BSA)). Association of 25 nM, 12.5 nM, 6.25 nM and 3.13 nM recombinant human gp130 with His-tag (Acro, ILT-H52H2) in KB were measured for 300 s followed by dissociation in KB for 300 s. The data were fitted and analyzed with ForteBio data analysis software 8.0 using a 1:1 binding model after Savitzky – Golay filtering if needed. Competition of the four paratopes in binding to the respective receptor were analyzed in an epitope binning experiment, where HIS1K biosensors were used to load 5 µg/mL human recombinant gp130-His (Acro, ILT-H52H2) for 180 s in PBS, followed by 60 s sensor rinsing in KB. Association of 200 nM first antibody in 300 s was combined with a second association (300 s) of another antibody in 200 nM with an included concentration spike of 100 nM of the first used antibody. KB+spike and KB only control values were measured in parallel to ensure visualization of additional association by alignment at 300 s. In each experiment, a negative control using an unrelated antibody and a baseline association in KB instead of the respective protein or antigen was included.

### 4.7 Thermal shift assay

Differential scanning fluorimetry (DSF) was used taking advantage of the fluorescence change of tryptophan residues within a protein during denaturation. 20 µl of 0.5 mg/ml protein solution was analyzed by heating the sample from 25°C to 95°C with a gradient of 1°C per minute using a NanoTemper Prometheus NT.48 nanoDSF device.

### 4.8 Retroviral transduction of Ba/F3 cells

Gene transfer by retroviral transduction is enabled by utilizing the virus producing cell line Phoenix Eco (Ursula Klingmüller, DKFZ, Heidelberg, Germany) and the target cell line Ba/F3. Phoenix cells were seeded and transfected with pMOWS plasmids containing a puromycin or hygromycin resistance and the gene of interest, following the manufactures protocol for TurboFect™ (Thermo Fisher Scientific, Waltham, USA, cat. #R0532). Medium was changed 6 h post transfection with DMEM containing 30% FCS and 1% P/S. The supernatant was collected 24 h later and filtered through a filter with 0.45 µm pore size (Carl Roth, Karlsruhe, Germany). 1x10^5^ Ba/F3 cells were added to 250 µl virus supernatant in a total volume of 300 µl, mixed with 8 µg/ml polybrene (#TR-1003-G, Merck, Darmstadt, Germany) and centrifuged 3 h at 1800 rpm and RT. Afterwards, the cells were resuspended in 5 ml growth medium and plated as usual. Hygromycin (#1287.2, Carl Roth, Karlsruhe, Germany) selection started one day post infection by adding 1 mg/ml into the medium. Puromycin (#0240.1 Carl Roth, Karlsruhe, Germany) selection was initiated 48 h post infection using 1.5 µg/ml. pMOWS-GFP vectors with either puromycin or hygromycin resistance were used as a positive control for transduction and selection, whether pEGFP was used as a positive control for Phoenix cell transfection but a negative control for the selection process of transduced Ba/F3 cells.

### 4.9 Proliferation assay

Ba/F3 cells are a controlled and sensitive system for analyzing gp130-mediated signaling, due to their dependence on cytokine stimulation for survival and proliferation. For the proliferation assay, Ba/F3 cell lines were washed 3 times with PBS to remove any cytokines. The cells were resuspended in DMEM (high glucose; Gibco, 41966-029) with 10% FCS and 1% P/S. Per well, 50.000 cells were seeded onto a 96-well plate and incubated for 72 h with the cytokine (1 ng/ml IL-11; 10 ng/ml IL-11:100 ng/ml sIL-11R; 1 ng/ml IL-6; 1 ng/ml Hyper-IL-6; 1 ng/ml OSM; 0.1 ng/ml LIF; 10 ng/ml CNTF; 5 ng/ml mIL27) and different concentrations (between 0.001 and 1000 nM) of the gp130 nanobodies as indicated in a total volume of 100 µl. To analyze cell proliferation, 20 μl Cell-Titer-Blue Reagent (Promega, Karlsruhe, Germany, cat. #G808A) is added to each well of the plate. Fluorescence is measured in the Tecan Infinite M200 PRO Reader (Tecan, Crailsheim, Germany) (Excitation 560 nm/Emission 590 nm) every 20 min for 2 h. The background signal at time-point zero is subtracted from the final fluorescence intensity. Data is presented as relative light units (RLU) and every condition is measured in triplicates. Error bars indicate standard deviation.

### 4.10 Stimulation assays

For the stimulation assay, the Ba/F3 cell lines were washed 5 times with PBS and starved for 5 h in DMEM^-/-^. The cells were divided into 1.5 ml reaction tubes, mixed with the indicated concentration of gp130 nanobodies and incubated for a further 30 min before being stimulated with the appropriate cytokine for a total of 20 min (20 ng/ml IL-11; 400 ng/ml IL-11:800 ng/ml sIL-11R; 20 ng/ml IL-6; 20 ng/ml Hyper-IL-6; 10 ng/ml OSM; 0.1 ng/ml LIF; 10 ng/ml CNTF; 10 ng/ml mIL27). For the stimulation assay, higher concentrations than used in the proliferation assays were applied, because the detection limit for Western blotting requires stronger stimulation. Cell supernatant containing Hyper-IL-6 (approx. 20 ng/ml) was used as a positive control (+). Unstimulated cells are indicated with (-) and serve as the negative control of each experiment. The cells were washed with PBS, lysed in 200 μl Lysis buffer (10 mM Tris-HCl, pH 7.8, 150 mM NaCl, 0.5 mM EDTA, 0.5% NP-40, 1 mM sodium vanadate, 10 mM MgCl_2_ and a cOmplete, EDTA-free protease inhibitor cocktail tablet (Roche Diagnostics, Mannheim, Germany)) and incubated at 4°C for 2 h. Cell debris was removed by centrifugation (14000 rpm, 20 min, 4°C) and the supernatant was collected for further analysis. Protein concentration was determined by BCA Protein Assay (Thermo Fisher Scientific, Waltham, USA), following the manufactures protocol. Lämmli samples were obtained by mixing the cell lysates with 6x Lämmli buffer and boiled at 95°C for 10 min.

### 4.11 Western blotting

50 µg total protein lysate, or 20 µl pull down sample, was used for Western blotting as described earlier (66). In brief, proteins were separated in a 10% SDS gel electrophoresis and blotted to a nitrocellulose membrane using the Trans-Blot Turbo Transfer System (Bio-Rad, Hercules, USA). Membranes were blocked with Intercept^®^ (TBS) Blocking Buffer (Lincoln, USA) and incubated with the primary antibody overnight in a dilution of 1:1000. The next day, membranes were washed 3 times with TBS-T (TBS+ 0.2% Tween20) and incubated with the secondary antibody for 1 h at room temperature in a dilution of 1:10,000. Blots were scanned using the LI-COR Odyssey^®^ XF Imaging System (Lincoln, USA) and evaluated with the Image Studio software version 5.2.

### 4.12 Intracellular STAT3/pSTAT3 staining

To quantify the phosphorylation state of STAT3 via FACS measurements, intracellular staining was performed after stimulation. Therefore, 1.3x10^5^ cells were seeded in V-bottom plate and centrifuged for 5 min at 300 xg and 4°C. All centrifugation steps are performed analogues. The supernatant was discarded and the cells were fixed in 2% PFA solution for 10 min at 37°C. The cells were centrifuged, the supernatant was removed and the cells were permeabilized with 90% ice cold methanol and incubated for 10 min at -20°C. Next, the cells were washed twice in wash buffer (1% BSA, 0.5 mM EDTA in PBS) and incubated with anti-pSTAT3^Y705^-Alexa647 (#557815) (1:200) (BD Biosciences, Franklin Lakes, NJ, USA) and anti-STAT3-PE (#560391) (1:50) (BD Biosciences, Franklin Lakes, NJ, USA) in a total volume of 50 µl at 4°C over night in 2% BSA, 0.5 mM EDTA in PBS. On the next day, cells were washed again twice and finally resuspended in 200 µl wash buffer. The fluorescence was determined in a FACSCanto II (BD Biosciences) flow cytometer and analyzed by FACS DIVA software (BD Biosciences).

### 4.13 Immunofluorescence based binding assay

Association of the nanobodies to a membrane-bound gp130 and variants was detected by immunofluorescence-based assay with Ba/F3 or HEK293T cells. Ba/F3 cells were either expressing gp130 or mgp130, transmitted by retroviral transduction as described earlier. 5x10^5^ cells per well were distributed in a V-bottom 96-well plate and washed with 200 µl PBS by centrifuging the plate at 1200 rpm for 5 min at room temperature (RT). All washing steps were carried out analogues. The pellet was resuspended in 100 µl PBS+1% BSA, mixed with 3 µg of the nanobodies or Hyper IL-6-Fc (3 µg for Ba/F3 mgp130 and 300 µg for Ba/F3 gp130) and incubated at RT for 30 min. Hyper IL-6-Fc serves as a positive control. Unstained cells were treated with neither primary (nanobodies) nor secondary antibody. As a negative control, cells were not incubated with any nanobodies, but with the secondary antibody: anti-human-Fc (#109-115-098, Jackson Immuno Research, Baltimore). Following the first incubation, cells were centrifuged and washed once with PBS. Afterwards, cells were resuspended in 50 µl PBS+1% BSA containing the secondary antibody in a dilution of 1:100. The plate was incubated for 30 min at 4°C in the dark. Washing was performed like before and the cells were finally resuspended in 200 µl PBS+1% BSA and used for FACS analysis with a FACS Canto II using FACSDiva software (BD Biosciences, Franklin Lakes, USA) in the PE channel. Data represents the mean of fluorescence in the PE channel using FlowJo V10 (FlowJo LLC, Ashland, USA). In order to determine the binding epitope of the nanobodies, the same technique was used as explained before but utilizing HEK cells for easier gene transmission. HEK293T cells were seeded in a density of 3,5x10^5^ cells per 6-well. 24 h later, cells were transfected with the gp130-WT-HA or deletion variant gp130-ΔD1-FLAG using Turbofect™ (Thermo Fisher, Waltham, USA) following manufactures protocol. After 48 h, 5x10^5^ cells/well were transferred to a 96-well V-bottom plate and treated with the nanobodies and secondary PE-labeled antibody as described before. The cells were analyzed by FACS and quantified by the mean of fluorescence in the PE channel.

### 4.14 Pull down

Prior performing the pull-down (PD) assay, HEK293T were transfected with cDNAs coding for gp130 and variants containing a HA-Tag, using Turbofect™ (Thermo Fisher, Waltham, USA) following manufactures protocol. After 48 h, cells were lysed in pull-down buffer (PD buffer: 50 mM Tris-HCl pH 7.5, 150 mM NaCl, 1 mM EDTA pH 8 and cOmplete™, EDTA-free Protease Inhibitor Cocktail (Roche, Mannheim, Germany)) containing 1% Triton-X100, for 1 h on ice and centrifuged 20 min at 14.000 rpm at 4°C. Supernatant was distributed in 1.5 ml tubes and incubated with 1 µg/ml nanobody over night at 4°C while slow mixing. 50 µl were taken as the input sample and mixed with 10 µl 6x Lämmli buffer. Protein A agarose beads (Roche, Mannheim, Germany) were washed with PD buffer three times and incubated over night at 4°C. The next day, 30 µl beads were added to each tube and incubated for 4 h at 4°C while slow mixing. The beads were washed three times by centrifuging at 5.000 xg for 30 sec, removing the supernatant and adding fresh PD buffer. After the final wash, the whole fluid was removed with an insulin syringe and the beads were resuspended in 60 µl 1x Lämmli buffer Lämmli samples were boiled at 95°C for 10 min and analyzed by western blotting.

### 4.15 Transmigration assay

The transmigration assay was performed following the protocol of Justus, Marie, Sanderlin et al. (67). HT-29 cells, a colorectal adenocarcinoma cell line, is known for their migratory and invasive behavior in *in vitro-* and xenograft models, which can also be IL-11 dependent (37, 68, 69). For the assay, HT-29 cells were trypsinized, counted and plated into a 0.8 µm pore size 24-well trans-well insert with a total cell number of 1x10^5^ cells in 100 µl McCoy’s 5A medium containing 0.1% FCS. Cells were mixed with 100 nM gp130 nanobody as indicated and pre-incubated for 10 min at 37°C. Subsequently, 600 µl migration buffer: McCoy’s 5A medium, 0.1% FCS and 100 ng/ml Hyper IL-11, was added to the 24-well plate. The medium must touch the trans-well membrane. The plate was incubated for 5 h in an incubator at 37°C with 5% CO_2._ Next, non-migrated cells were removed from the top of the membrane with a cotton swab. Remaining cells on the lower side of the membrane were fixed with 70% ethanol for 15 min at RT. The membranes were dried under an airflow and stained in 0.2% crystal violet solution for 5 min at RT. Excess staining reagent was removed by three washing steps with PBS. The membranes were dried once more and imaged using a Keyence BZ-9000 microscope with a 2x objective. The migrated cells were quantified by ImageJ software. Results are displayed as total particle area. Possible impurities were quantified separately and subtracted from the total number.

### 4.16 Statistical analyses

Proliferation assay data are shown from one representative experiment out of at least three independent assays yielding consistent results. IC_50_ values were calculated using nonlinear regression with a variable slope model in GraphPad Prism 8.0 (version 8.0.2 for Windows; GraphPad Software, www.graphpad.com), based on data from three separate experiments. Results are expressed as mean ± standard deviation (SD). For direct comparisons, unpaired two-tailed t-tests were performed (GraphPad Prism 8.0.2, GraphPad Software Inc). Statistical significance was defined as p < 0.05 (*p < 0.05; **p < 0.01; ***p < 0.001).

## Data Availability

The original contributions presented in the study are included in the article/**Supplementary Material**. Further inquiries can be directed to the corresponding author.
