# Supplementary material for: Targeting the major pro-inflammatory interleukin-6-type cytokine receptor gp130 by antagonistic single domain antibodies
Source: Front Immunol. 2025 Aug 15;16:1613004. doi: 10.3389/fimmu.2025.1613004 (PMC12394151; doi:10.3389/fimmu.2025.1613004)
Supplement: Supplementary file 1 [file Table1.docx]

Supplementary Material

Targeting the major pro-inflammatory Interleukin-6-type cytokine receptor gp130 by antagonistic single domain antibodies

Silke Pudewell^1,#^, Julia Heuser^1,#^, Inna Dorogobed^2^, Britta Lipinski^3^, Thi Hong Hue Tran^3^, Pia Metzenmacher^1^, Richard Kunze^1^, Felix Geyer^2^, Stefan Zielonka^3^, Doreen M. Floss^1^, Jens Moll^1^, Harald Kolmar^2^, Jürgen Scheller^1,*^

^1^Institute of Biochemistry and Molecular Biology II, Medical Faculty, Heinrich-Heine-University, 40225 Düsseldorf, Germany.

^2^Applied Biochemistry, Institute for Organic Chemistry and Biochemistry, Technical University of Darmstadt, 64289 Darmstadt, Germany.

^3^Biomolecular Immunotherapy, Institute for Organic Chemistry and Biochemistry, Technical University of Darmstadt, 64287 Darmstadt, Germany.

^#^ contributed equally

*corresponding author: Jürgen Scheller, jscheller@hhu.de


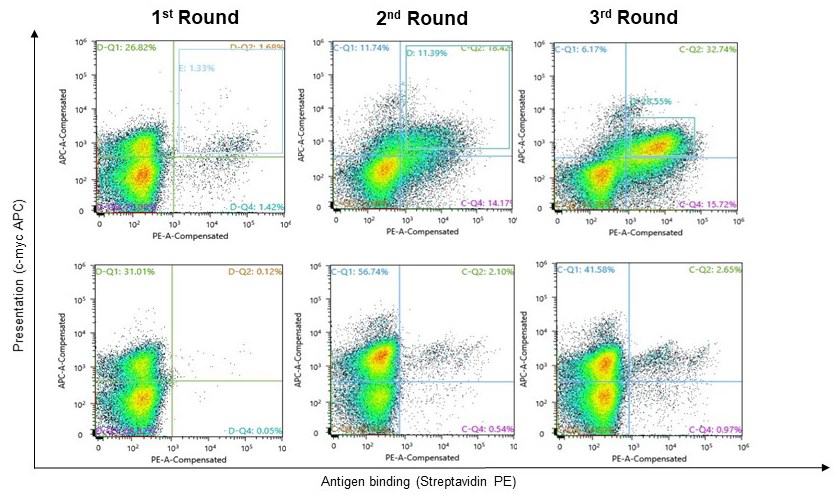


250 nM
sgp130

250 nM
sgp130

250 nM
sgp130

**(B)**

**(A)**

**Figure S1. Fluorescence activated cell sorting and alignment of all seven VHH sequences.** (A) Three enrichment rounds were performed with cells expressing gp130 VHHs using 250 nM biotinylated sgp130 (upper row). The negative control without antigen is presented in the bottom row. Nanobody-presenting cells are shifted to APC (anti-c-myc; Y-axis) and PE (streptavidin; X-axis) positive populations. Double positive cells were selected for the next round. (B) The sequences of GP01, GP06, GP11, GP12, GP13, GP14 and GP20 are presented in an alignment. Blue marking resembles identical amino acids, orange background marks amino acid differences.


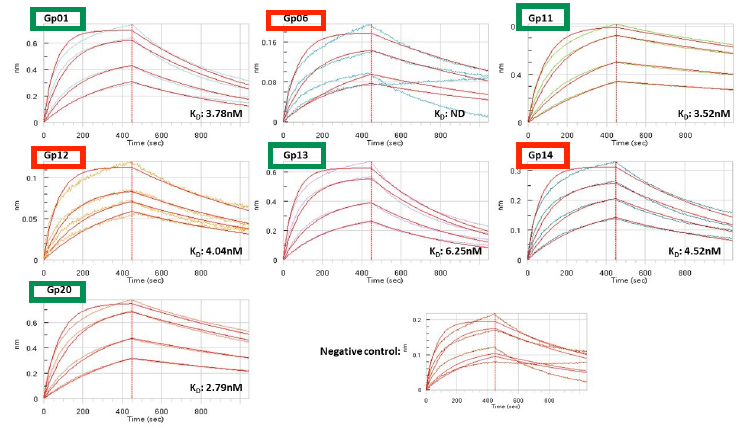


**Figure S2. Interaction of gp130 with the VHHs.** For affinity determination via biolayer interferometric measurements, the Octet RED96 system (ForteBio, Sartorius, Göttingen, Germany) was used. Anti-human IgG-Fc capture (AHC, Sartorius, Göttingen, Germany) biosensors were soaked in PBS pH 7.4 for at least 10 min before the start of the assay and subsequently used for the immobilization of the VHH-Fc variants. An association step using concentrations of gp130, ranging from 6.25 nM to 400 nM was performed followed by a dissociation step in buffer. Data analysis was performed using ForteBio data analysis software. Binding kinetics, including the equilibrium constant K_D_, were determined using Savitzky–Golay filtering and a 1:1 Langmuir binding model. The negative control included PBS only and displayed an increase of layer thickness of about 0.2 nm, which could also be detected for the fusion proteins GP06-Fc, GP12-Fc and GP14-Fc. The nanobodies GP01-Fc, GP11-Fc, GP13-Fc and GP20-Fc increased layer thickness to over 0.6 nm and are therefore considered binders. The equilibrium dissociation constants (K_D_) revealed single-digit nanomolar affinity of GP01-Fc (3.78 nM), GP11-Fc (3.52 nM), GP13-Fc (6.25 nM) and GP20-Fc (2.79 nM) towards gp130.

**
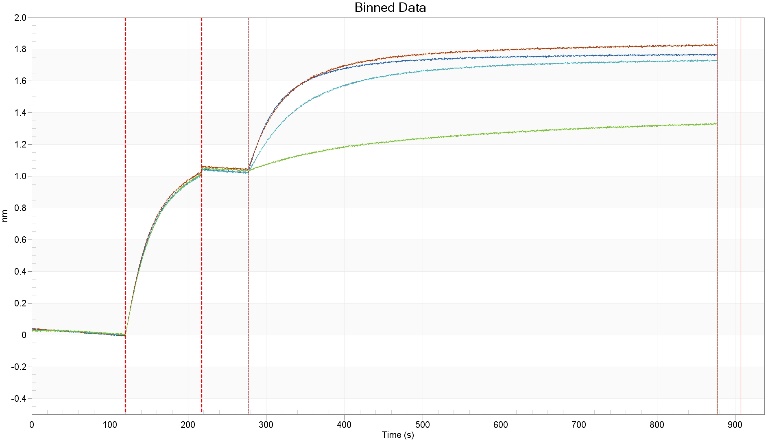

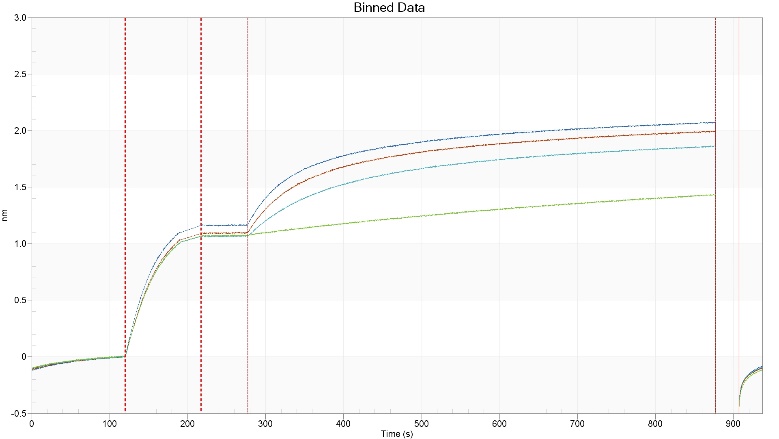

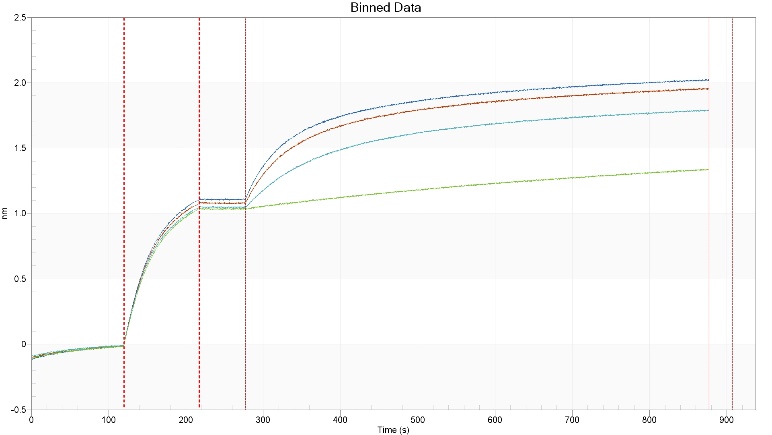

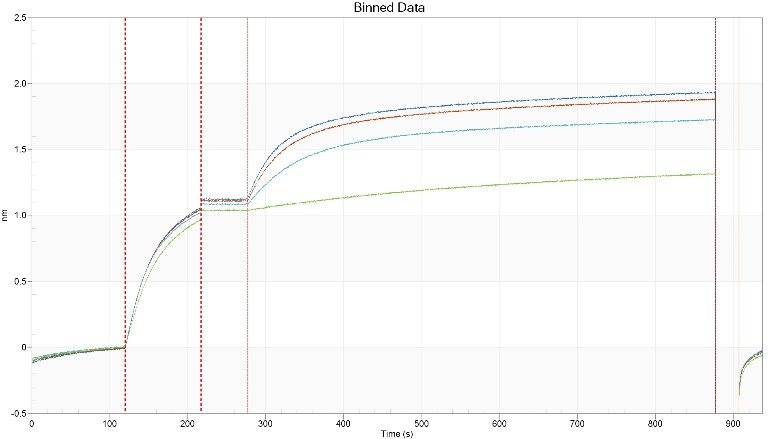
Figure S3. Original BLI data of Figure 2A.** 100 nM of the gp130 extracellular domain was preincubated with 0 nM (dark blue), 50nM (brown), 100 nM (light blue) or 500 nM (green) Hyper-IL-11 and the binding to the precoated gp130 specific nanobodies was measured.

GP20-Fc

GP13-Fc

GP11-Fc

GP01-Fc

**
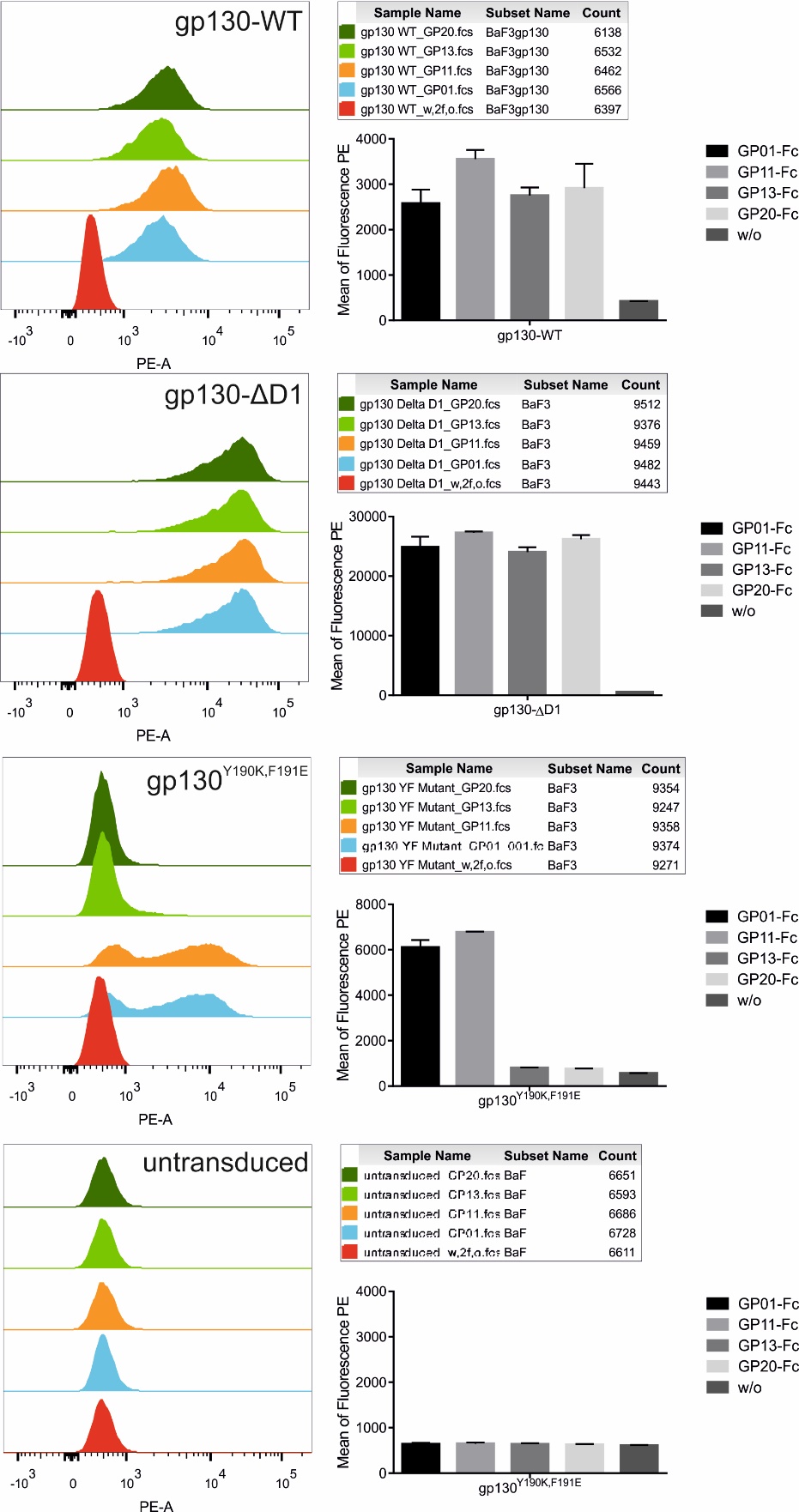
**

**Figure S4. Binding assay of the nanobodies to gp130-WT, -ΔD1 and -Y190K, F191E variants.** Stable transduced Ba/F3 cells with gp130-WT, gp130-ΔD1 or gp130^Y190K, F191E^ in a binding assay with no nanobody (red), GP01-Fc (blue), GP11-Fc (orange), GP13-Fc (light green) and GP20-Fc (dark green), incubated with PE-labeled anti-Fc antibody. Bar charts represent mean of fluorescence in the PE channel ± SD (n=3). The histograms are one representative out of the replicates.


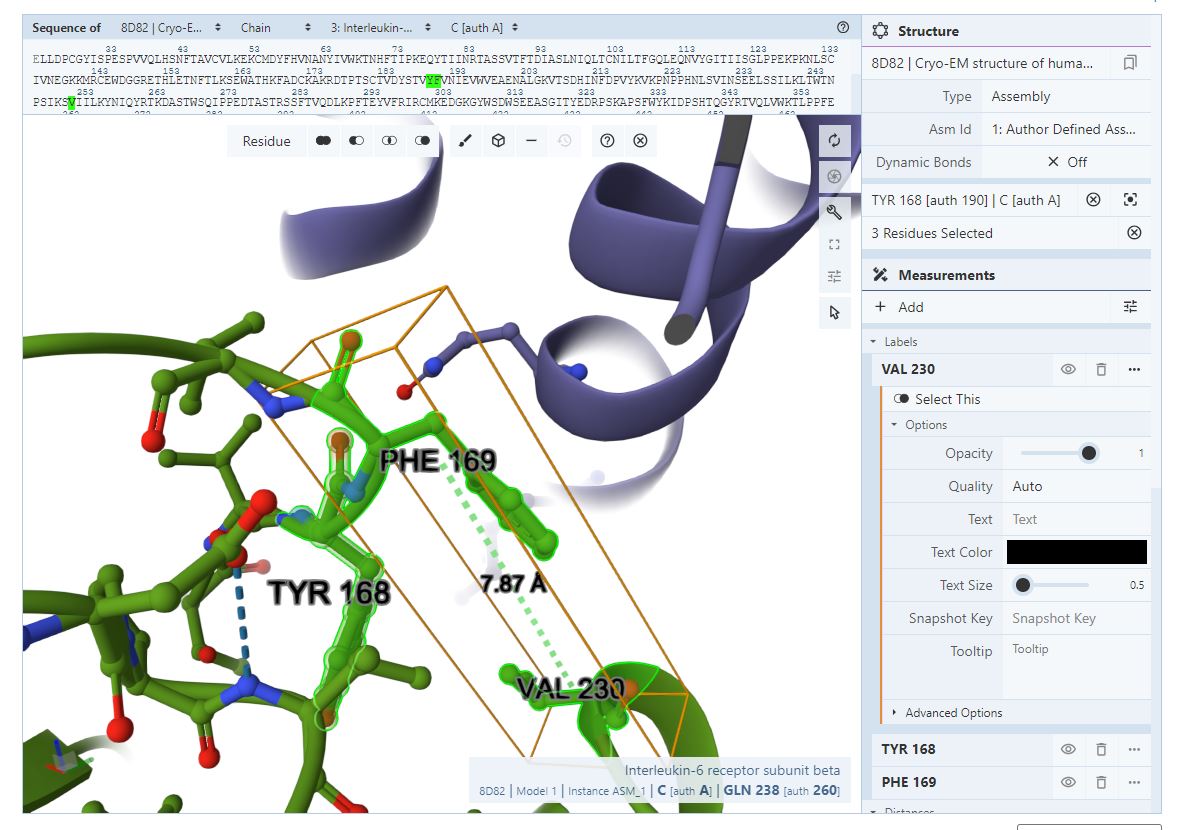

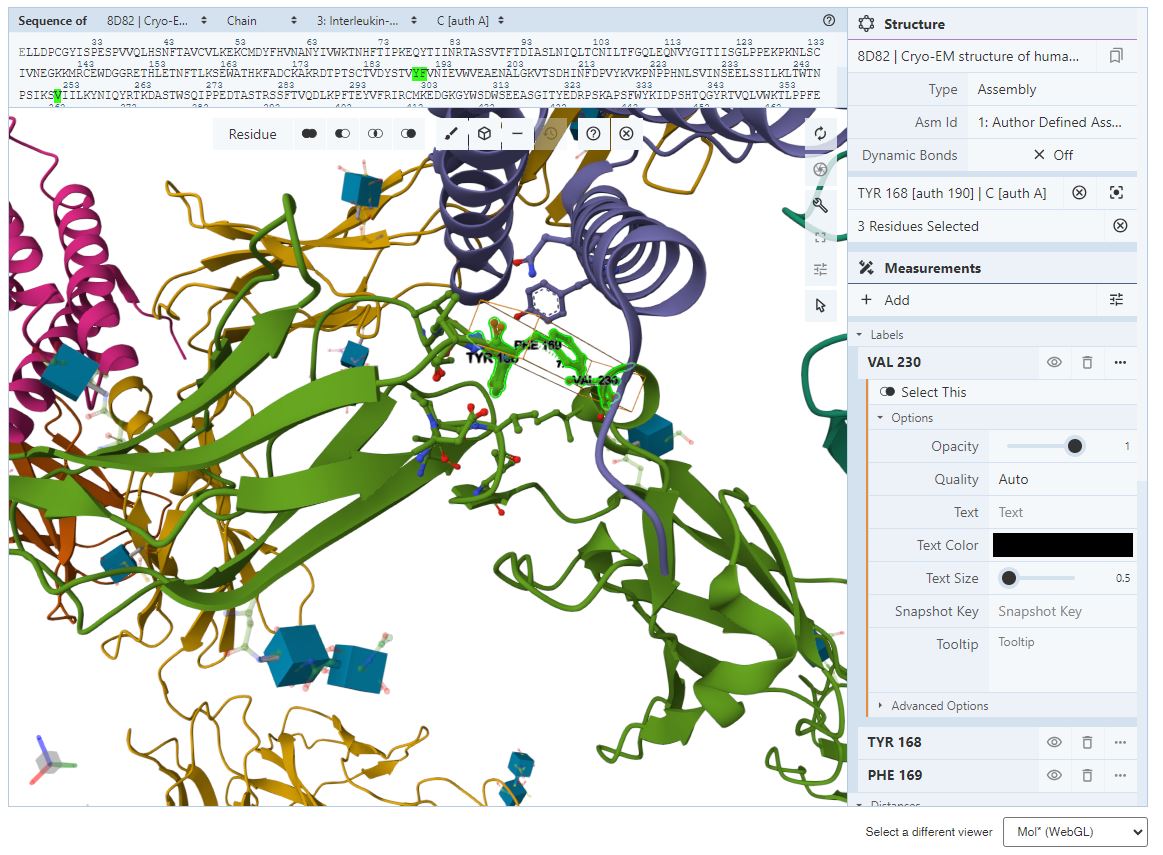


**Figure S5. Crystal structures of gp130 in contact to IL-6:IL-6R.** D2/D3 contact sites in gp130 includes F191 (F169) in the EF-loop of D2 and V252 (V230) in the D3 domain. PDB: 8D82 – Zhou et al. *Sci. Adv.* **2023**. [1]


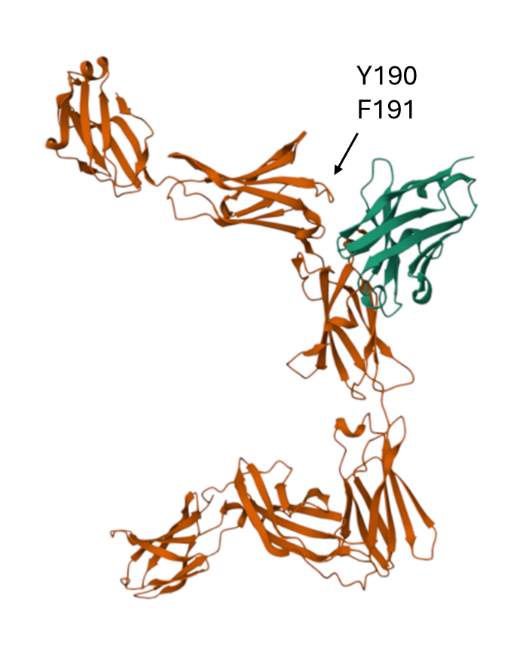

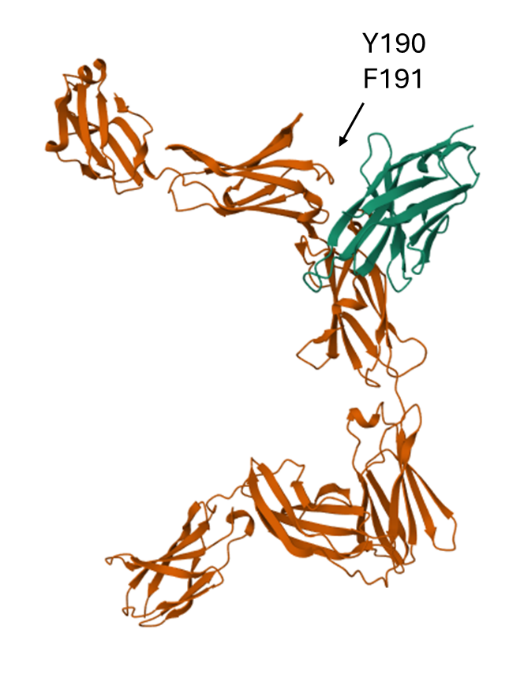

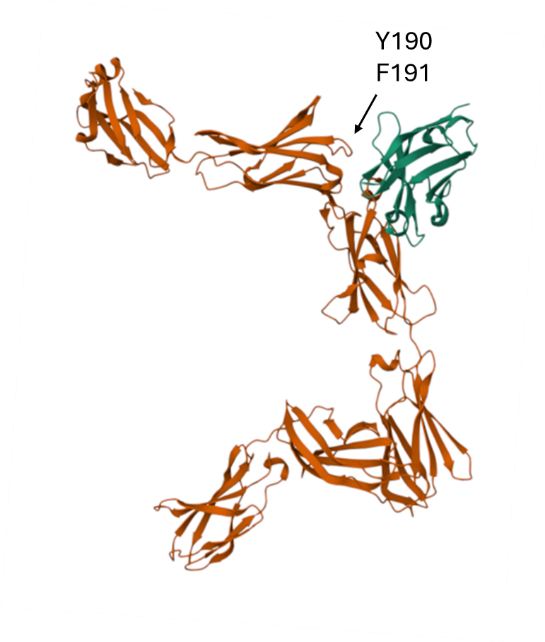

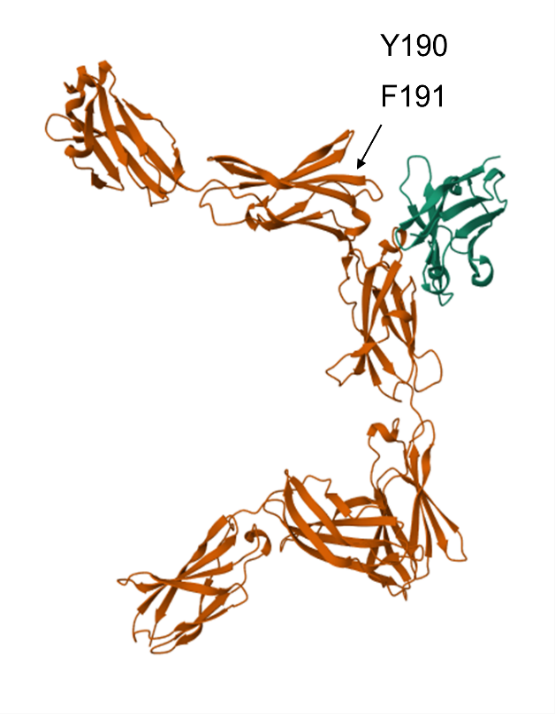


**(A)**

**GP01**

**(B)**

**(C)**

**(D)**

**GP11**

**GP13**

**GP20**

**Figure S6. Docking analysis.** All four nanbodies GP01 (A) GP11 (B) GP13 (C) and GP20 (D) are predicted to bind to the same epitope, which is located at the transition from domain 2 to domain 3. The overall pLDDT score is high for all predicted nanobody:antigen complexes. The CDR loops partially have only moderate confidence for prediction. The binding appears to be mainly contributed by the CDR3 loop and the CC' loop located in the framework region 2. Overall, all CDR loops seem to be involved in binding. With respect to Y190 and F191 located in the connecting EF loop, there is only a direct interaction predicted for F191, whereas Y190 is mainly involved in intramolecular interactions. Interestingly, F191 has more interactions with the nanobodies GP13 and GP20, as W118 in FR4 is predicted to be involved in an interaction and is in a radius of 4 angstroms. For all four nanobodies an interaction between F191 and Y37 and R45 is predicted, where the additional interaction with W118 is only predicted for GP13 and GP20. In contrast, for GP01 and GP11, Y190 and F191 are located a little further away and are therefore less involved in intermolecular interactions. Prediction was performed by AlphaFold 3 and ColabDock. [2,3]


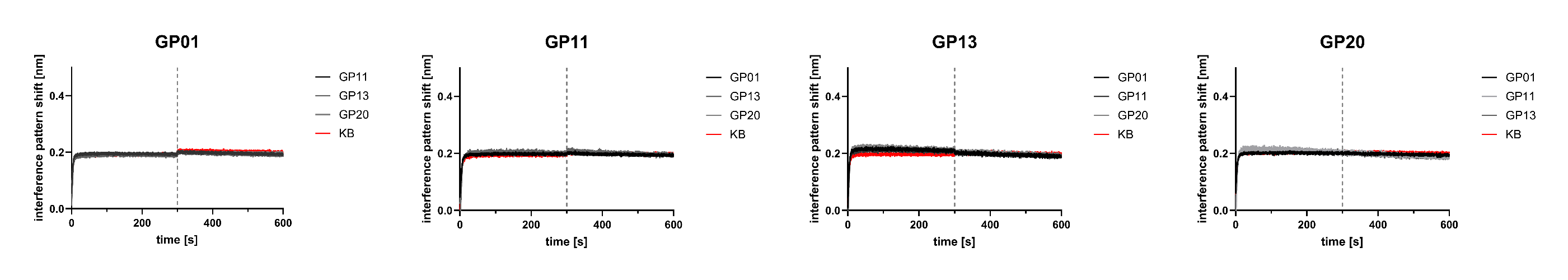

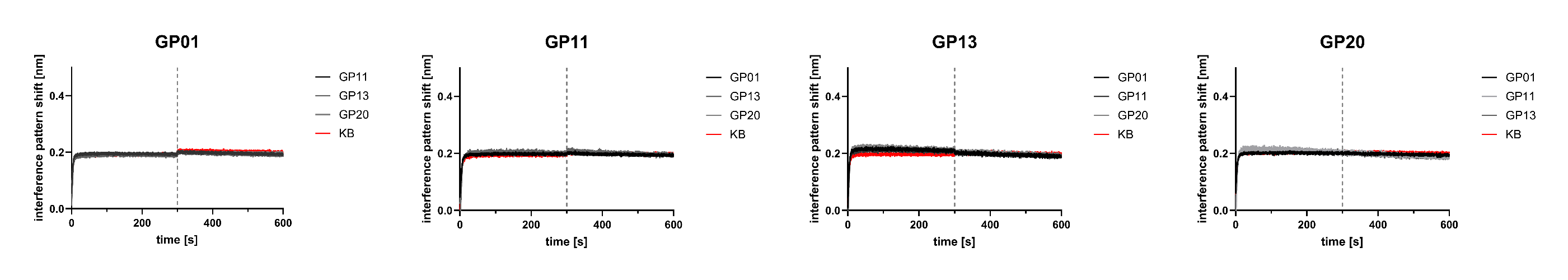


**Figure S7. Epitope Binning assay of GP01-Fc, GP11-Fc, GP13-Fc and GP20-Fc with gp130-His.** Competition of the four paratopes in binding to the respective receptor were analyzed in an epitope binning experiment using the Octet RED96 system (ForteBio, Pall Life Science). HIS1K biosensors were used to load 5 µg/mL recombinant human gp130-His (Acro, ILT-H52H2) for 180 s in PBS, followed by 60 s sensor rinsing in kinetic buffer (KB; PBS + 0.1% Tween-20 and 1% bovine serum albumin (BSA)). Association of 200 nM first antibody in 300 s was combined with a second association (300 s) of another antibody in 200 nM with an included concentration spike of 100 nM of the first used antibody. KB+spike and KB only control values were measured in parallel to ensure visualization of additional association by alignment at 300 s. In each experiment, a negative control using an unrelated antibody and a baseline association in KB instead of the respective protein or antigen was included.


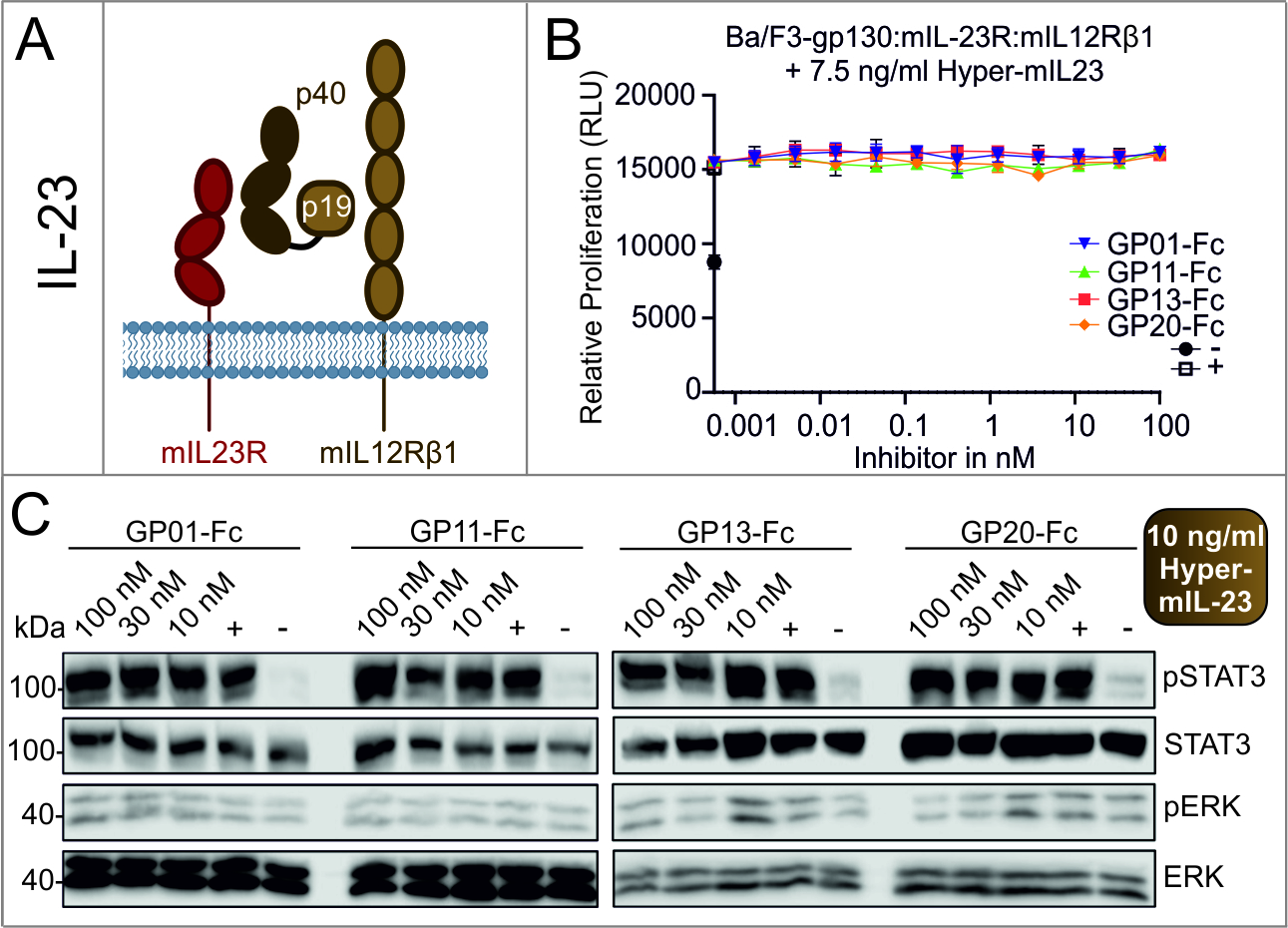


**Figure S8. IL-23 proliferation and stimulation assays. (**A) IL-23 signaling was analyzed with Ba/F3 cells expressing gp130, murine IL-23R and murine IL-12Rβ1. (B) Proliferation was induced by 7.5 ng/ml murine Hyper-IL-23 (fusion protein of p40 and p19), and could not be inhibited by the gp130 nanobodies. (C) STAT3 and ERK phosphorylation was checked by stimulating the cells with 10 ng/ml Hyper-mIL-23. The positive control (+) was stimulated with Hyper-IL-6 and the negative control (-) displays unstimulated cells. The VHH-Fc fusion proteins did not inhibit the phosphorylation of pSTAT3.

**References:**

1. Zhou, Y.; Stevis, P.E.; Cao, J.; Saotome, K.; Wu, J.; Zaretsky, A.G.; Haxhinasto, S.; Yancopoulos, G.D.; Murphy, A.J.; Sleeman, M.W.; et al. Structural insights into the assembly of gp130 family cytokine signaling complexes. *Sci. Adv.* **2023**, *9*, doi:10.1126/sciadv.ade4395.

2. Abramson, J.; Adler, J.; Dunger, J.; Evans, R.; Green, T.; Pritzel, A.; Ronneberger, O.; Willmore, L.; Ballard, A.J.; Bambrick, J.; et al. Accurate structure prediction of biomolecular interactions with AlphaFold 3. *Nat. 2024 6308016* **2024**, *630*, 493–500, doi:10.1038/s41586-024-07487-w.

3. Feng, S.; Chen, Z.; Zhang, C.; Xie, Y.; Ovchinnikov, S.; Gao, Y.Q.; Liu, S. Integrated structure prediction of protein–protein docking with experimental restraints using ColabDock. *Nat. Mach. Intell. 2024 68* **2024**, *6*, 924–935, doi:10.1038/s42256-024-00873-z.
